# Understanding willingness and barriers to participate in clinical trials during pregnancy and lactation: findings from a US study

**DOI:** 10.1186/s12884-024-06710-w

**Published:** 2024-07-26

**Authors:** Melanie H. Jacobson, Emily Yost, Shirley V. Sylvester, Cheryl Renz, Diego F. Wyszynski, Kourtney J. Davis

**Affiliations:** 1 Global Epidemiology Organization, Janssen Research & Development, LLC, 1125 Trenton-Harbourton Road, Titusville, NJ 08560 US; 2 Global Public Health, Johnson & Johnson, Zug, Switzerland; 3 Pregistry, LLC, Los Angeles, CA US; 4 Pregistry, LLC, London, England

**Keywords:** Pregnancy, Lactation, Clinical trial participation

## Abstract

**Background:**

Due to the exclusion of pregnant and lactating people from most clinical trials, there is an incomplete understanding of the risks and benefits of medication use in these populations and therapeutic decision-making is often conducted without adequate evidence. To change this paradigm, it is imperative to understand the perspectives of pregnant and lactating individuals concerning their participation in clinical trials.

**Objectives:**

To describe attitudes, perceptions, barriers, and preferences of pregnant and postpartum people in the United States (US) regarding participation in clinical trials and to identify factors influencing participation.

**Methods:**

In November 2022, individuals aged ≥ 18 residing in the US who self-identified as pregnant or pregnant within the last 12 months were invited to complete an online survey about their perspectives regarding clinical trial participation. The survey included questions about demographic characteristics, health history, behaviors, and willingness to participate in clinical trials while pregnant and/or lactating. Multivariable logistic regression models were fit to identify predictors of clinical trial participation.

**Results:**

Among the 654 respondents, 34.8% and 40.9% reported being likely or extremely likely to participate in a clinical trial for a new medication while pregnant or lactating, respectively; and 24.5% and 41.7% for a new vaccine while pregnant or lactating, respectively. Higher educational attainment (≥ Bachelor’s degree) was associated with greater likelihood of clinical trial participation in pregnancy (odds ratio (OR) = 1.50, 95% Confidence Interval (CI): 1.01, 2.25 for medications; OR = 2.00, 95% CI: 1.28, 3.12 for vaccines). Chronic medical conditions were associated with a greater likelihood of participation in clinical trials for vaccines during lactation (OR = 1.59, 95% CI: 1.07, 2.36). The most cited motivator for participation in a clinical trial while pregnant or lactating was anticipated personal medical benefit (85.8% and 75.6%, respectively), while the primary deterrent was possible risk to the fetus or baby (97.9% and 97.2%, respectively).

**Conclusions:**

Willingness of a US sample to participate in clinical trials while pregnant or lactating varied by demographics and health status, with safety to the fetus being a nearly universal concern. These findings have implications for enhancing inclusion of pregnant and lactating people in clinical research and developing effective and equitable recruitment strategies.

**Supplementary Information:**

The online version contains supplementary material available at 10.1186/s12884-024-06710-w.

## Introduction

Over 3.6 million people in the United States give birth annually and over 80% breastfeed their infants [1, 2]. These births occur in generally healthy people and also those affected by serious illnesses such as hypertension, diabetes, asthma, mental health disorders, autoimmune disorders, cancers and other medical conditions that require chronic or urgent treatment during pregnancy. Up to 94% of pregnant people take one or more prescription medications for chronic illnesses or for acute conditions that arise during pregnancy [3, 4], and despite a sparse literature on postpartum medication use, over half of lactating individuals take at least one medication [5, 6]. Nonetheless, most medical prescribing information have little to no human data about the benefits and risks of use during pregnancy and are limited to nonclinical reproductive and developmental toxicity data. This leads to therapeutic decision-making with limited information and “off-label” use [7, 8]. Similarly, the general absence of data on the extent of the transfer of specific drugs into human milk, and other factors, make health care providers cautious when advising on medication use while breastfeeding [9].

This disparity in evidence exists because pregnant and lactating people have historically been excluded from most clinical trials. The Institute of Medicine issued a report nearly 30 years ago on the challenges and barriers of including women in clinical research that recommended pregnant people be presumed eligible for participation and that Institutional Review Boards (IRBs) exclude pregnant participants only when there was no prospect of medical benefit and a risk of significant harm to the offspring was known or could be plausibly inferred [10]. Over the last decade, many multi-stakeholder initiatives and workshops sponsored by the United States Food and Drug Administration, National Institutes of Health, Task Force on Research Specific to Pregnancy Women and Lactating Women, and National Academies of Sciences, Engineering, and Medicine aimed to promote and overcome challenges for including pregnant and lactating people in clinical research [11–14]. Resulting from this theoretical consensus were recommendations and solutions that have been shared to increase inclusion and provide guidance through such measures as tailored approaches to trial designs [15]. However, currently, pregnant people continue to be excluded from the vast majority of pharmacological therapeutic and preventive vaccine trials. This default exclusion is consequential from ethical, health care delivery, and equitability perspectives; as was recently underscored by the COVID-19 pandemic, which left pregnant people without evidence on both prevention and treatments at the time of Emergency Use Authorizations despite their disproportionately higher risk for disease-related complications and unmet need.

Despite these directives to increase inclusion, motivators and deterrents of participation from the pregnant or lactating person’s perspective are not well understood. While it is known that clinical trials involving pregnant people face enrollment and engagement challenges, such as a greater reluctance in taking any investigational medication or vaccine during gestation and perceived potential negative consequences on the fetus, a comprehensive assessment of the causes of these recruitment challenges is lacking [16]. Most of the studies that have been conducted on this topic focus on trials for pregnancy- or obstetric-specific indications, such as for hypothetical vaccines for Group B streptococcus [17], respiratory syncytial virus [18], or Zika virus [19] or medications for preeclampsia treatment or prevention [20, 21]. However, a few studies reported in the literature have collected data on attitudes about general clinical trial participation in pregnancy, though none explicitly focused on participation while lactating or breastfeeding [22–24]. One of these, a large study recently conducted in the US, sought to identify factors that influenced women’s participation in clinical research [22]. However, their derived motivator and deterrent scales were not thematically organized, making interpretation of findings difficult; and because they included both pregnant and non-pregnant women, the factors that were queried were more generic. Notwithstanding, substantial literature exists based on in-depth, semi-structured interviews and focus groups with qualitative analyses to understand perspectives of pregnant and lactating individuals and to generate aggregate themes underlying decisions about participation across a range of actual or hypothetical clinical trial designs [16, 19, 25, 26]. Recently, a mixed-methods systematic review was conducted on this topic that established the need for a multi-tiered approach to increase inclusion based on the complex and multi-tiered system in which research participation takes place, taking into account the pregnant or lactating individual’s perspectives, her familial and personal relationships, her relationships with healthcare staff and the healthcare system, and the larger forces at play in the healthcare and research ecosystems [27]. We leveraged this body of work to inform the design of a quantitative study in a US sample to (1) identify factors associated with participation in clinical trials during both pregnancy and lactation and (2) collect in-depth data on motivators and deterrents of participation. We note that our study was intended to have a wide scope by querying perspectives on participation in clinical trials for both medications and vaccines, during both pregnancy and lactation.

## Methods

This cross-sectional study was based on a quantitative survey administered online between November 7 and November 21, 2022. The study included individuals in the United States who were either pregnant or had been pregnant within the past 12 months, were aged 18 years or older, and possessed at least an eighth-grade level of conversational proficiency in English. Participation in the study was voluntary and responses were collected anonymously.

A pre-test of the questionnaire was conducted with a small number of volunteers and feedback was obtained and incorporated as needed. Approval to conduct the study was obtained from the US-based WIRB-Copernicus Group (WCG) IRB. Given that this was a fully anonymous study with no collection of personally identifiable data, it was classified as exempt by the WCG IRB under the regulations at 45 CFR 46.104(d)(2). All participants provided informed consent prior to study participation, and no financial compensation was provided.

Participants were recruited through social media platforms, primarily Facebook and Instagram, with targeted English-language advertising campaigns aimed at pregnant and postpartum individuals (Supplemental Figure). Advertisements included direct and situational prompts related to thoughts, feelings, and opinions about clinical trial conduct in pregnancy and/or lactation. When potential participants clicked the links in these advertisements, they were brought to a screen to sign the consent and take the survey, which included information about the research objectives and standards of confidentiality regarding the use of the data. The survey contained questions on demographics, pregnancy history, lactation, chronic medical conditions, medication use, self-rated health, and knowledge about and willingness to participate in clinical trials during pregnancy and lactation. When asked about clinical trials, the survey specified that this was referring to interventional research studies performed in volunteers that test and evaluate new medicines, vaccines, or medical devices [28]. The survey design incorporated skip patterns to ensure efficiency and relevance, directing participants to relevant sections based on their eligibility. The survey instrument is available in the Supplemental Material.

### Statistical analysis

The primary outcome of interest was willingness to participate in clinical trials. This was examined in four iterations as willingness to participate in: (1) a clinical trial for a medication during pregnancy, (2) a clinical trial for a vaccine during pregnancy, (3) a clinical trial for a medication while breastfeeding, and (4) a clinical trial for a vaccine while breastfeeding. For the questions concerning trials for medications, individuals with chronic medical conditions were asked about participation regarding treating these, and those who did not have any were asked to imagine they did. Individuals who were currently pregnant or were postpartum at the time of survey administration, but reported not having breastfed for at least two weeks after delivery, were asked only about willingness to participate in trials during pregnancy; individuals who were postpartum and reported having breastfed for at least two weeks after delivery were asked about willingness to participate in trials during pregnancy and while breastfeeding. While willingness to participate in trials was queried on a five-point Likert scale ranging from extremely unlikely to extremely likely, we collapsed ‘likely’ and ‘extremely likely’ into one category and compared it with those who endorsed any of the other three (extremely unlikely, unlikely, and neutral/not sure). Participant characteristics were assessed across strata of willingness to participate and chi-squared and t-tests were used to test for statistical differences. Multivariable logistic regression models were fit to estimate mutually adjusted associations between participant characteristics and willingness to participate in order to identify predictors of clinical trial participation. Models were stratified by the type of trial (i.e., medications or vaccines) as well as the timeframe (i.e., pregnancy or breastfeeding). In supplemental analyses, we examined the impact of reducing the model by removing covariates that were measuring similar constructs (i.e., education and income) and deriving a composite of race and ethnicity, but because estimates and measures of precision did not materially change, we present full model results.

**Fig. 1:**
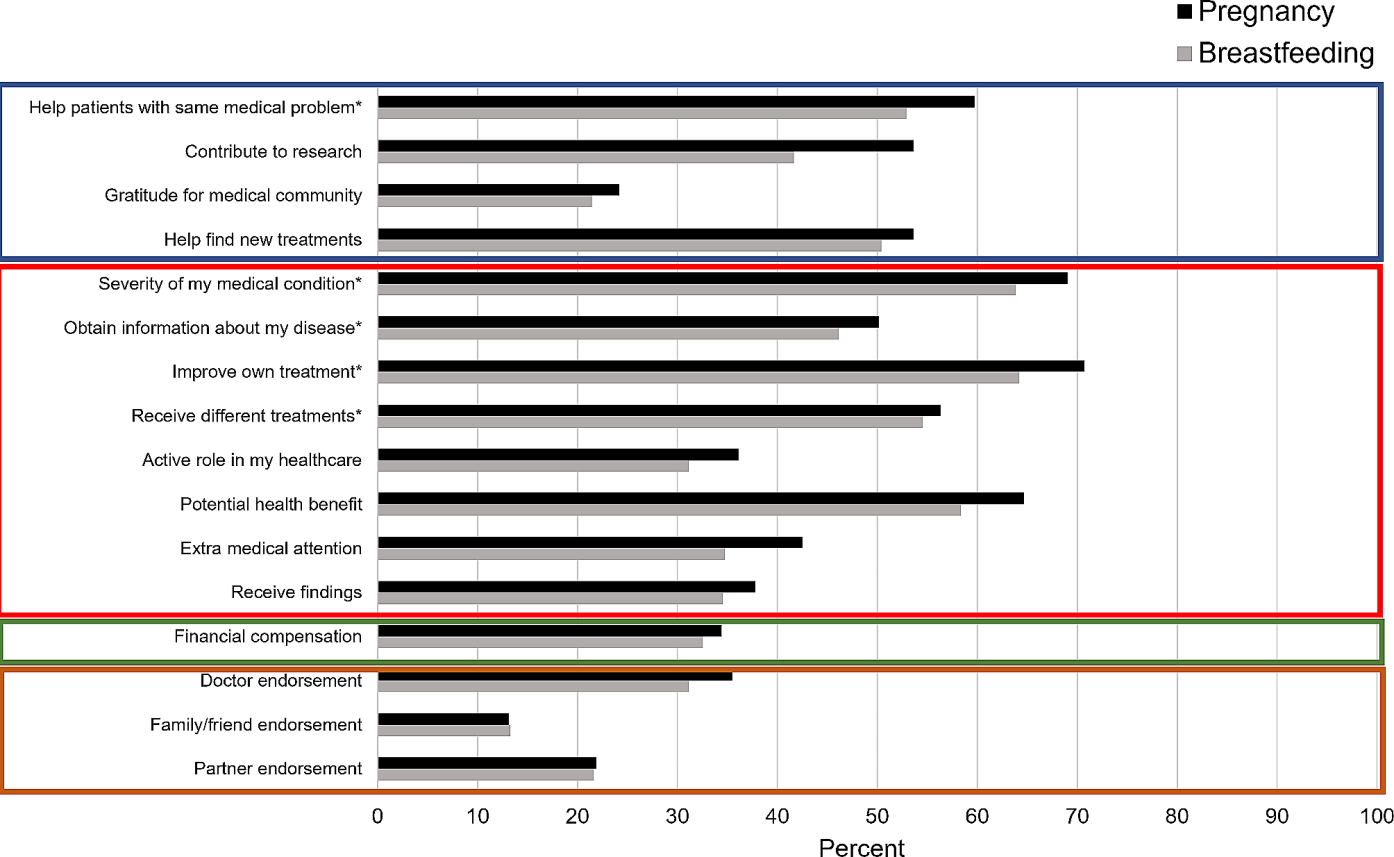
Percent individuals who reported very or extreme importance for items in altruism (blue), personal medical benefit (red), financial (green), and support network approval (orange) domains as motivators for clinical trial participation. Items marked with an asterisk denote items that were only asked among those with chronic medical conditions

**Fig. 2:**
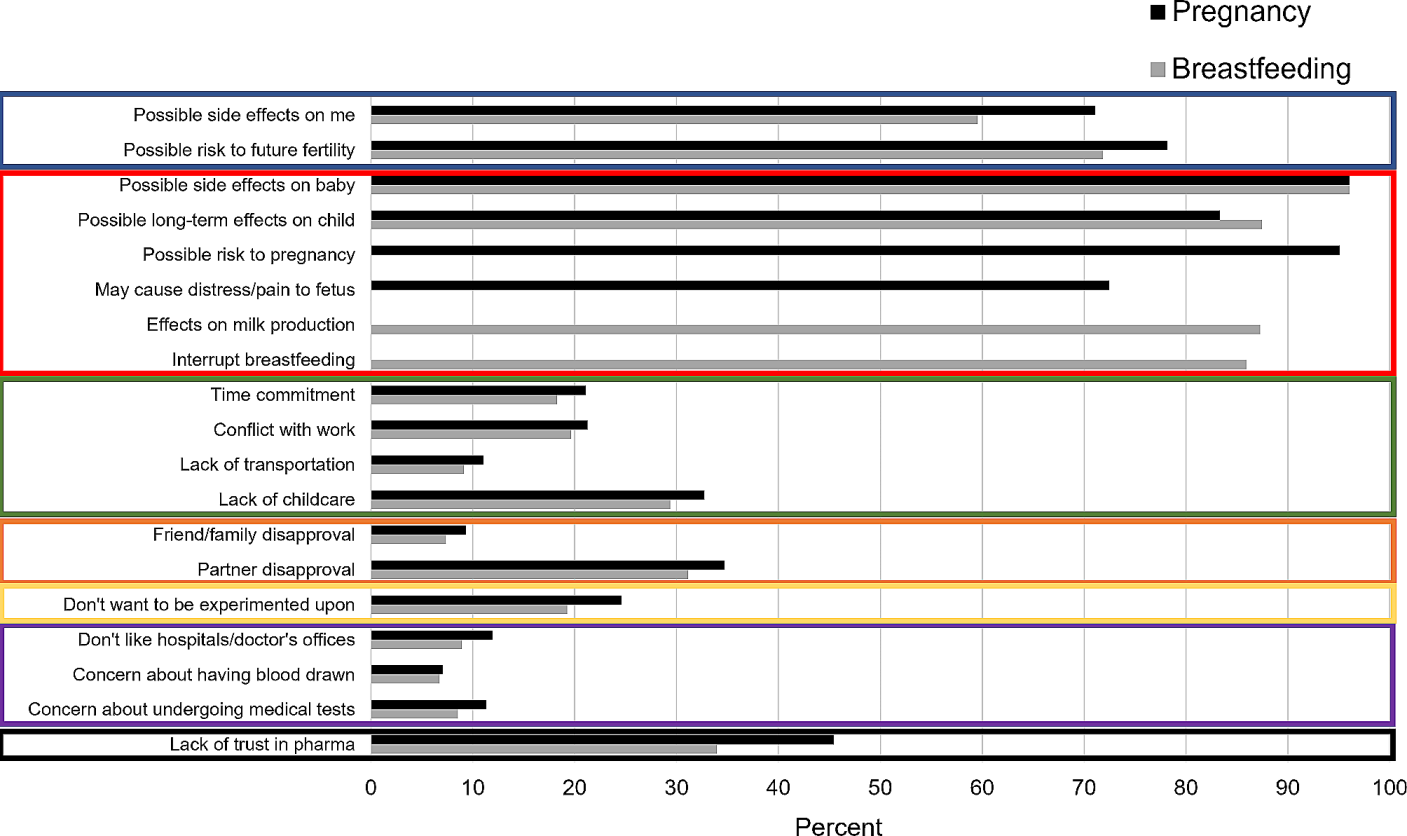
Percent individuals who reported very or extreme importance for items in safety for oneself (blue), safety for fetus/baby (red), logistics challenges (green), support network disapproval (orange), discomfort with experimentation (yellow), dislike of medical interventions (purple), and distrust in pharmaceutical companies (black) domains as deterrents for clinical trial participation

Potential motivators and deterrents of participation were also examined. First, the 16 motivators and 19 deterrents were classified into domains based on thematic clustering of factors (Figs. 1 and 2). We evaluated internal consistency within domains using Cronbach’s alpha, which were deemed to have met an acceptable level of agreement if they had an α > 0.70 [29]. The criterion for fulfilling a domain was based on whether at least one constituent motivator or domain was endorsed as at least ‘very important’ (from a five-point Likert scale ranging from ‘not at all important’ to ‘very important’). Sociodemographic predictors of motivator and deterrent domain endorsement were then assessed using multivariable logistic models similar to those described above. Lastly, these domains were examined in relation to the willingness to participate in medication or vaccine trial in pregnancy or while breastfeeding as the outcomes by simultaneously including them in multivariable logistic regression models along with the original set of participant characteristic covariates. The deterrent domain related to safety concerns for the fetus or baby was not considered in analytic models as an exposure or outcome variable due to its nearly complete endorsement (> 97%).

Finally, in exploratory analyses, we examined participant characteristics across strata of prior participation in clinical trials and level of familiarity with clinical trials.

Associations were evaluated by statistical significance at α = 0.05.

## Results

A total of 654 individuals completed the survey, the majority (79.8%) of whom were postpartum (i.e., pregnant within the previous twelve months) at that time (Table 1). Among them, nearly all (97.9%) reported that they breastfed for at least two weeks after delivery. The sample was relatively demographically homogenous with 83.9% self-identifying as white and 90.6% as non-Hispanic. There was more heterogeneity by educational attainment and household income: 48.2% had at least a Bachelor’s degree and 63.0% had a household income of less than $90,000. The average age was 31.4 years (standard deviation = 5.1). The majority of participants resided in suburban locations, with approximately equal proportions residing in rural or urban settings, with representation from across 49 states. Self-report of a chronic medical condition was common (63.8%), and of those, 62.4% reported taking a medication to manage it. Despite this, 94.5% rated their general health as good, very good, or excellent. The majority (69.8%) of participants had been pregnant before and 45.2% had a history of non-live birth (e.g., miscarriage, ectopic pregnancy, stillbirth). There was a substantial degree of willingness to participate in clinical trials during pregnancy: 35% were likely to participate in a medication trial and 25% were likely to participate in a vaccine trial. Overall, willingness to participate was greater while breastfeeding (41% for medication trials and 42% for vaccine trials) (Supplemental Table 1).

**Table 1:** Survey participant characteristics by likelihood of participation in a clinical trial for a medication or vaccine in pregnancy (*n* = 654)

	Overall		Participation in a clinical trial for a medication^a^					Participation in a clinical trial for a vaccine				
			Likely (*n* = 225, 35%)		Not likely (422, 65%)			Likely (*n* = 160, 25%)		Not likely (*n* = 494, 76%)		
Characteristic	N	%	N	%	N	%	p	N	%	N	%	p
Pregnancy status							0.04					0.29
Pregnant	132	20.2	55	24.4	75	17.8		37	23.1	95	19.2	
Postpartum	522	79.8	170	75.6	347	82.2		123	76.9	399	80.8	
Breastfeeding behavior (among postpartum only)							0.12					0.76
Ever breastfed	509	97.9	163	96.5	341	98.6		119	97.5	390	98.0	
Never breastfed	11	2.1	6	3.6	5	1.5		3	2.5	8	2.0	
Age (years)							0.26					0.78
18–24	72	11	19	8.4	51	12.1		15	9.4	57	11.5	
25–29	152	23.2	54	24.0	96	22.8		36	22.5	116	23.5	
30–34	247	37.8	81	36.0	166	39.3		60	37.5	187	37.9	
35–44	183	28.0	71	31.6	109	25.8		49	30.6	134	27.1	
Mean (SD)	31.4	5.1	31.7	4.9	31.3	5.2	0.37	31.7	5.2	31.4	5.1	0.54
Race							0.36					0.14
White	547	83.9	182	81.3	359	85.3		129	81.1	418	84.8	
Black	24	3.7	13	5.8	11	2.6		10	6.3	14	2.8	
American Indian/Alaska Native	6	0.9	3	1.3	3	0.7		3	1.9	3	0.6	
Asian	20	3.1	8	3.6	12	2.9		7	4.4	13	2.6	
Pacific Islander	2	0.3	0	0.0	2	0.5		0	0.0	2	0.4	
Other	23	3.5	9	4.0	14	3.3		3	1.9	20	4.1	
Multiracial	30	4.6	9	4.0	20	4.8		7	4.4	23	4.7	
Ethnicity							0.38					0.69
Non-Hispanic	579	90.6	196	89.1	376	91.3		141	89.8	438	90.9	
Hispanic	60	9.4	24	10.9	36	8.7		16	10.2	44	9.1	
Education							0.92					< 0.01
≤High school	92	14.1	31	13.8	60	14.2		17	10.6	75	15.2	
Some college or trade school	247	37.8	81	36.0	162	38.4		48	30.0	199	40.3	
Bachelor’s degree	178	27.2	64	28.4	113	26.8		44	27.5	134	27.1	
Postgraduate	137	21.0	49	21.8	87	20.6		51	31.9	86	17.4	
Household income							< 0.01					0.03
Prefer not to answer or don’t know	30		7		23			5		25		
<$10,000-<$40,000	161	25.8	61	28.0	98	24.6		35	22.6	126	26.9	
≥$40,000-<$90,000	232	37.2	81	37.2	148	37.1		51	32.9	181	38.6	
≥$90,000-<$150,000	155	24.8	38	17.4	116	29.1		40	25.8	115	24.5	
≥$150,000	76	12.2	38	17.4	37	9.3		29	18.7	47	10.0	
Urbanicity							0.01					< 0.01
Rural	109	16.7	33	14.7	74	17.5		16	10.0	93	18.8	
Suburban	421	64.4	135	60.0	282	66.8		102	63.8	319	64.6	
Urban	124	19.0	57	25.3	66	15.6		42	26.3	82	16.6	
Marital status							0.11					0.86
Married or cohabitating	607	92.8	204	90.7	397	94.1		148	92.5	459	92.9	
Single	47	7.2	21	9.3	25	5.9		12	7.5	35	7.1	
Chronic medical condition							0.66					0.45
No	237	36.2	85	37.8	152	36.0		54	33.8	183	37.0	
Yes	417	63.8	140	62.2	270	64.0		106	66.3	311	63.0	
Gravidity							0.10					0.21
Primigravida	196	30.2	58	26.0	135	32.2		54	34.2	142	28.9	
Multigravida	453	69.8	165	74.0	284	67.8		104	65.8	349	71.1	
History of non-live birth							0.63					0.36
No	355	54.8	119	53.6	233	55.6		91	58.0	264	53.8	
Yes	293	45.2	103	46.4	186	44.4		66	42.0	227	46.2	
Worked in healthcare							0.94					0.69
No	407	62.4	141	62.7	262	62.4		102	63.8	305	62.0	
Yes	245	37.6	84	37.3	158	37.6		58	36.3	187	38.0	
Prior participation in clinical trial							< 0.01					< 0.01
No	552	85.9	174	78.7	374	90.1		118	74.7	434	89.5	
Yes	91	14.2	47	21.3	41	9.9		40	25.3	51	10.5	
General health							0.68					0.99
Poor or fair	36	5.5	12	5.4	24	5.7		8	5.0	28	5.7	
Good	213	32.6	77	34.4	133	31.5		52	32.5	161	32.7	
Very Good	236	36.1	74	33.0	159	37.7		59	36.9	177	35.9	
Excellent	168	25.7	61	27.2	106	25.1		41	25.6	127	25.8	

^a^Excludes *n* = 7 individuals with missing responses

All statistical tests were conducted with α = 0.05

Willingness to participate in clinical trials for new medications or vaccines in pregnancy varied by participant characteristics (Table 1). Those who were currently pregnant, with household incomes of ≥$150,000, living in urban settings, and had participated in a clinical trial prior were more likely to be willing to participate in a clinical trial for a new medication in pregnancy. Trends were similar for vaccine trials in pregnancy, though those with a postgraduate degree were also more willing than those with lower educational attainment (*p* < 0.01). Patterns were similar across strata of willingness to participate in clinical trials while breastfeeding, though income was not associated, single (vs. married) individuals were more willing to participate in medication trials, and those with chronic medical conditions (vs. without) were more willing to participate (Supplemental Table 1).

Adjusted associations from multivariable logistic models between participant characteristics and willingness to participate in clinical trials are shown in Tables 2 and 3. Education and urbanicity were the two variables that maintained a multivariable-adjusted association with willingness to participate in medication or vaccine clinical trials during pregnancy (Table 2). For example, those with at least a college degree were more likely to be willing to participate in medication (odds ratio (OR) = 1.50, 95% Confidence Interval (CI): 1.01, 2.25) or vaccine (OR = 2.00, 95% CI: 1.28, 3.12) trials in pregnancy compared with those with less education. Those living in urban (vs. suburban or rural) settings were more likely to be willing to participate in clinical trials for medications (OR = 1.78, 95% CI: 1.16, 2.73) or vaccines (OR = 1.97, 95% CI: 1.25, 3.11) in pregnancy. Results were similar when examining willingness to participate in clinical trials during breastfeeding and there was an additional notable finding: those with chronic medical conditions (vs. not) were more likely to be willing to participate in a clinical trial for a vaccine while breastfeeding (OR = 1.59, 95% CI: 1.07, 2.36) (Table 3).

**Table 2:** Adjusted odds ratios for the likelihood to participate in a clinical trial for a medication or vaccine in pregnancy (*n* = 654)

	Likely or extremely likely to participate in clinical trial during pregnancy vs. not			
Variable	For a medication		For a vaccine	
	OR	95% CI	OR	95% CI
Age (1 year increase)	1.02	0.98, 1.06	1.01	0.96, 1.05
White vs. Non-White	0.88	0.54, 1.43	0.85	0.50, 1.44
Hispanic vs. Non-Hispanic	1.28	0.70, 2.34	1.23	0.64, 2.40
Currently pregnant vs. not	1.42	0.92, 2.17	1.25	0.78, 2.01
≥College degree vs. < College	1.50	1.01, 2.25	2.00	1.28, 3.12
≥$90,000 household income vs. <$90,000	0.71	0.47, 1.07	1.13	0.73, 1.76
Chronic medical condition vs. healthy	0.86	0.60, 1.23	1.12	0.75, 1.67
Married/cohabitating vs. single	0.82	0.41, 1.63	0.87	0.40, 1.91
Urban vs. rural or suburban	1.78	1.16, 2.73	1.97	1.25, 3.11
Primigravida vs. multigravida	0.78	0.52, 1.18	1.12	0.72, 1.72

All statistical tests were conducted with α = 0.05

**Table 3:** Adjusted odds ratios for the likelihood to participate in a clinical trial for a medication or vaccine while breastfeeding (*n* = 504)

	Likely or extremely likely to participate in clinical trial while breastfeeding vs. not			
Variable	For a medication		For a vaccine	
	OR	95% CI	OR	95% CI
Age (1 year increase)	0.99	0.95, 1.03	0.99	0.95, 1.04
White vs. Non-White	1.16	0.65, 2.08	1.18	0.66, 2.11
Hispanic vs. Non-Hispanic	1.29	0.64, 2.62	1.82	0.90, 3.66
≥College degree vs. < College	2.28	1.46, 3.56	2.19	1.41, 3.40
≥$90,000 household income vs. <$90,000	0.75	0.47, 1.17	1.03	0.66, 1.61
Chronic medical condition vs. healthy	1.29	0.87, 1.91	1.59	1.07, 2.36
Married/cohabitating vs. single	0.58	0.24, 1.41	0.77	0.32, 1.88
Urban vs. rural or suburban	1.84	1.11, 3.04	1.69	1.02, 2.79
Primigravida vs. multigravida	0.75	0.49, 1.14	1.09	0.72, 1.65

All statistical tests were conducted with α = 0.05

Among the 654 study participants, 91 (14.2%) had previously participated in a clinical trial with 37.4% of them during pregnancy and/or breastfeeding (Supplemental Table 2). Those who participated prior (vs. not) were more likely to self-identify as Black, American Indian, or Asian, have household incomes of ≥$90,000, reside in either rural or urban settings (i.e., not suburban), and have experience working in healthcare. Most reported having moderate (42%) or substantial (44%) familiarity with clinical trials, defined as knowing “some facts” and “having a good understanding”, respectively (Supplemental Table 3). Those with more familiarity with clinical trials were more likely to be postpartum, older, have more education and household income, not have a chronic medical condition, have experience working in healthcare, and better self-rated health.

Potential motivators for participating in clinical trials in pregnancy or while breastfeeding were categorized into four domains: altruism, personal medical benefit, financial incentive, and support network approval (Fig. 1 and Supplemental Table 4). Deterrents were categorized into seven domains: concerns about safety for oneself, concerns about safety for the fetus or baby, logistics challenges, support network disapproval, discomfort with experimentation, dislike of medical interventions, and distrust in pharmaceutical companies (Fig. 2 and Supplemental Table 4). Measures of internal consistency within derived domains generally showed good performance (α > 0.70), though safety concerns for oneself were slightly suboptimal (α = 0.56 and 0.51 for pregnancy and breastfeeding, respectively) (Supplemental Table 5). The most common motivator domain was personal medical benefit followed by altruism, whereas the most common deterrent domain was safety concerns for the fetus or baby followed by safety concerns for oneself.

Table 4 shows the predictors of motivator and deterrent domain endorsement generated from multivariable models. Those with chronic medical conditions (vs. without) were more likely to be motivated by personal medical benefit (OR = 3.24, 95% CI: 2.00, 5.24), altruism (OR = 1.82, 95% CI: 1.26, 2.63), and less likely to be motivated by financial compensation (OR = 0.60, 95% CI: 0.41) to participate in trials in pregnancy, with similar estimates while breastfeeding. Those who had at least a college degree (vs. less than) and those who were older were also less likely to be motivated by financial compensation (OR = 0.51, 95% CI: 0.35, 0.79 and OR = 0.93, 95% CI: 0.90, 0.97, respectively). Hispanic (vs. non-Hispanic) individuals and those with chronic medical conditions were less likely to be deterred by safety concerns for oneself (OR = 0.32, 95% CI: 0.16, 0.67 and OR = 0.56, 95% CI: 0.31, 0.99, respectively). Those who self-identified as white (vs. non-white) (OR = 0.59, 95% CI: 0.36, 0.95), had greater household incomes (OR = 0.67, 95% CI: 0.45, 1.00), or had chronic medical conditions (OR = 0.68, 95% CI: 0.48, 0.97) were less likely to be deterred by logistical challenges when considering clinical trial participation in pregnancy specifically. Those with greater educational attainment (vs. less) were less likely to be deterred by discomfort with experimentation (OR = 0.61, 95% CI: 0.39, 0.95), dislike of medical interventions (OR = 0.45, 95% CI: 0.27, 0.74), and distrust in pharmaceutical companies (OR = 0.48, 95% CI: 0.33, 0.71) to participate in trials during pregnancy, with similar patterns shown for while breastfeeding. Those with chronic medical conditions were also less likely to be deterred by distrust in pharmaceutical companies (OR = 0.57, 95% CI: 0.40, 0.81).

**Table 4:** Associations between participant characteristics and domains of motivators and deterrents for participation clinical trials in pregnancy or while breastfeeding

	Motivators																	
	Personal medical benefit (*n*=85.8%)			Altruism (69.9%)			Financial compensation (34.4%)			Personal medical benefit (*n*=85.8%)								
**In pregnancy**	**OR**	**95% CI**		**OR**	**95% CI**		**OR**	**95% CI**		**OR**	**95% CI**							
Age (1 year increase)	1.00	0.95	1.06	0.99	0.95	1.03	0.93	0.90	0.97	0.99	0.96	1.03						
White vs. Non-White	0.69	0.34	1.40	0.57	0.32	1.00	0.63	0.38	1.06	1.10	0.68	1.76						
Hispanic vs. Non-Hispanic	0.80	0.35	1.80	1.13	0.58	2.20	1.18	0.63	2.22	1.22	0.68	2.17						
Currently pregnant vs. not	1.01	0.55	1.86	1.05	0.66	1.67	1.40	0.89	2.20	0.94	0.62	1.42						
≥College degree vs. < College	0.59	0.34	1.03	1.24	0.82	1.89	0.52	0.35	0.79	0.89	0.61	1.30						
≥$90,000 household income vs. <$90,000	1.06	0.60	1.85	0.99	0.65	1.52	0.55	0.36	0.87	1.06	0.72	1.56						
Chronic medical condition vs. healthy	3.24	2.00	5.24	1.82	1.26	2.63	0.60	0.41	0.88	1.14	0.81	1.60						
Married/cohabitating vs. single	0.93	0.33	2.58	1.16	0.57	2.37	0.71	0.36	1.40	1.11	0.57	2.16						
Urban vs. rural or suburban	0.60	0.34	1.08	1.28	0.79	2.08	1.21	0.77	1.92	0.98	0.64	1.48						
Primigravida vs. multigravida	1.42	0.81	2.49	1.03	0.68	1.55	0.69	0.45	1.06	1.30	0.89	1.89						
	Personal medical benefit (78.6%)			Altruism (62.1%)			Financial compensation (32.5%)			Support network endorsement (40.9%)								
**While breastfeeding**	**OR**	**95% CI**		**OR**	**95% CI**		**OR**	**95% CI**		**OR**	**95% CI**							
Age (1 year increase)	0.96	0.92	1.01	0.98	0.94	1.02	0.92	0.88	0.97	0.97	0.93	1.01						
White vs. Non-White	0.91	0.46	1.81	0.59	0.32	1.08	0.43	0.23	0.78	0.87	0.49	1.54						
Hispanic vs. Non-Hispanic	1.23	0.52	2.94	0.81	0.40	1.64	1.07	0.51	2.24	0.98	0.48	1.98						
≥College degree vs. < College	1.23	0.73	2.09	1.22	0.78	1.89	0.49	0.31	0.79	0.87	0.57	1.35						
≥$90,000 household income vs. <$90,000	0.99	0.57	1.70	1.16	0.73	1.83	0.78	0.47	1.30	1.26	0.80	1.97						
Chronic medical condition vs. healthy	2.93	1.84	4.67	1.63	1.10	2.42	0.86	0.56	1.32	1.39	0.94	2.07						
Married/cohabitating vs. single	0.72	0.23	2.30	0.54	0.20	1.42	0.58	0.24	1.40	1.74	0.68	4.45						
Urban vs. rural or suburban	0.68	0.38	1.22	1.17	0.69	1.99	1.25	0.73	2.16	1.55	0.94	2.55						
Primigravida vs. multigravida	1.29	0.77	2.16	1.14	0.75	1.75	0.66	0.41	1.05	1.12	0.74	1.69						
	***Deterrents***																	
	Safety concerns for oneself (87.6%)			Logistics (47.1%)			Support network disapproval (35.8%)			Uncomfortable with experimentation (24.6%)			Dislike medical interventions (19.9%)			Distrust in pharmaceutical companies (45.4%)		
**In pregnancy**	**OR**	**95% CI**		**OR**	**95% CI**		**OR**	**95% CI**		**OR**	**95% CI**		**OR**	**95% CI**		**OR**	**95% CI**	
Age (1 year increase)	0.99	0.94	1.04	1.01	0.97	1.04	0.96	0.93	1.00	1.00	0.96	1.04	1.02	0.98	1.07	1.03	1.00	1.07
White vs. Non-White	0.67	0.31	1.47	0.59	0.36	0.95	0.85	0.52	1.39	0.68	0.40	1.14	0.58	0.33	1.01	0.61	0.38	1.00
Hispanic vs. Non-Hispanic	0.32	0.16	0.67	1.43	0.78	2.61	0.70	0.37	1.32	0.94	0.49	1.82	0.87	0.42	1.77	1.07	0.59	1.95
Currently pregnant vs. not	1.29	0.67	2.49	0.78	0.51	1.20	1.22	0.79	1.88	0.86	0.53	1.40	1.04	0.62	1.75	0.87	0.57	1.34
≥College degree vs. < College	1.63	0.90	2.96	0.79	0.54	1.16	0.95	0.64	1.41	0.61	0.39	0.95	0.45	0.27	0.74	0.48	0.33	0.71
≥$90,000 household income vs. <$90,000	1.04	0.56	1.94	0.67	0.45	1.00	0.93	0.62	1.41	0.96	0.61	1.52	1.01	0.60	1.68	0.66	0.44	0.98
Chronic medical condition vs. healthy	0.56	0.31	0.99	0.68	0.48	0.97	0.80	0.56	1.14	0.86	0.58	1.27	0.74	0.49	1.14	0.57	0.40	0.81
Married/cohabitating vs. single	1.54	0.65	3.66	0.83	0.43	1.63	1.75	0.82	3.74	0.60	0.30	1.20	0.77	0.36	1.64	0.98	0.50	1.93
Urban vs. rural or suburban	1.14	0.59	2.20	1.54	1.01	2.36	0.65	0.41	1.03	0.78	0.47	1.28	0.80	0.46	1.37	0.90	0.58	1.38
Primigravida vs. multigravida	1.33	0.71	2.48	0.81	0.55	1.18	1.42	0.96	2.10	1.15	0.74	1.78	1.02	0.62	1.66	0.84	0.57	1.23
	Safety concerns for oneself (82.9%)			Logistics (41.5%)			Support network disapproval (31.9%)			Uncomfortable with experimentation (19.3%)			Dislike medical interventions (15.3%)			Distrust in pharmaceutical companies (33.9%)		
**While breastfeeding**	**OR**	**95% CI**		**OR**	**95% CI**		**OR**	**95% CI**		**OR**	**95% CI**		**OR**	**95% CI**		**OR**	**95% CI**	
Age (1 year increase)	1.00	0.94	1.05	1.02	0.98	1.07	0.98	0.94	1.03	1.00	0.95	1.06	1.02	0.96	1.08	1.00	0.96	1.04
White vs. Non-White	0.93	0.44	1.97	0.72	0.41	1.26	0.86	0.47	1.57	0.74	0.37	1.47	0.49	0.24	0.99	0.87	0.48	1.58
Hispanic vs. Non-Hispanic	0.37	0.17	0.80	1.36	0.68	2.72	0.77	0.36	1.67	0.70	0.28	1.73	0.83	0.33	2.08	0.91	0.44	1.87
≥College degree vs. < College	0.83	0.48	1.47	1.22	0.79	1.89	0.90	0.57	1.42	0.69	0.40	1.19	0.45	0.24	0.84	0.42	0.26	0.66
≥$90,000 household income vs. <$90,000	0.98	0.55	1.74	0.76	0.48	1.19	1.00	0.62	1.59	0.90	0.51	1.60	1.32	0.70	2.49	0.93	0.57	1.51
Chronic medical condition vs. healthy	0.50	0.29	0.87	0.73	0.50	1.08	0.89	0.59	1.34	0.46	0.29	0.75	0.72	0.42	1.23	0.48	0.32	0.73
Married/cohabitating vs. single	1.85	0.67	5.15	0.75	0.31	1.81	2.36	0.77	7.24	1.31	0.42	4.10	0.87	0.27	2.74	1.11	0.45	2.73
Urban vs. rural or suburban	1.34	0.67	2.68	1.56	0.95	2.57	0.85	0.49	1.46	0.88	0.46	1.69	0.78	0.38	1.61	1.23	0.72	2.08
Primigravida vs. multigravida	1.64	0.93	2.90	1.02	0.68	1.55	1.49	0.97	2.28	0.90	0.53	1.53	0.92	0.51	1.66	0.80	0.51	1.26

All statistical tests were conducted with α=0.05

Finally, in models mutually adjusted for all motivator and deterrent domains as well as participant characteristics, those who cited altruism were consistently more likely to participate in medication and vaccine trials across pregnancy and breastfeeding (ORs ranged from 3.88 to 4.50; Table 5 and Supplemental Table 6). Those motivated by personal medical benefit were specifically more likely to be willing to participate in a clinical trial for a vaccine in pregnancy (OR = 2.36, 95% CI: 1.06, 5.26). Those deterred by safety concerns for oneself, discomfort with experimentation, and distrust in pharmaceutical companies were all less likely to be willing to participate in any trials during pregnancy or breastfeeding (ORs ranged from 0.36 to 0.55, 0.23–0.56, and 0.27–0.52, respectively).

**Table 5:** Associations between motivator and deterrent domain endorsement and likelihood of participation in clinical trials in pregnancy or while breastfeeding

	Likely or extremely likely to participate in clinical trial during pregnancy vs. not							Likely or extremely likely to participate in clinical trial while breastfeeding vs. not					
	For a medication			For a vaccine				For a medication			For a vaccine		
*Motivators*	OR	95% CI		OR	95% CI			OR	95% CI		OR	95% CI	
Personal medical benefit	1.47	0.77	2.80	2.36	1.06	5.26		1.19	0.62	2.27	1.46	0.77	2.78
Altruism	3.88	2.33	6.47	4.02	2.13	7.56		4.50	2.64	7.69	4.00	2.38	6.74
Financial compensation	1.21	0.79	1.87	1.13	0.69	1.83		2.09	1.24	3.54	1.40	0.83	2.37
Support network endorsement	1.26	0.85	1.85	1.14	0.74	1.75		1.19	0.74	1.94	1.50	0.93	2.43
*Deterrents*													
Safety concerns for oneself	0.47	0.26	0.84	0.36	0.20	0.67		0.39	0.21	0.72	0.55	0.30	1.00
Logistics	0.82	0.55	1.23	1.08	0.69	1.69		1.01	0.63	1.63	0.90	0.56	1.45
Support network disapproval	0.76	0.49	1.18	1.02	0.62	1.67		0.79	0.46	1.35	0.89	0.52	1.54
Uncomfortable with experimentation	0.56	0.33	0.97	0.25	0.12	0.52		0.41	0.19	0.88	0.23	0.10	0.53
Dislike medical interventions	1.03	0.58	1.83	0.96	0.48	1.92		0.78	0.35	1.70	1.42	0.63	3.20
Distrust in pharmaceutical companies	0.52	0.34	0.79	0.46	0.28	0.74		0.35	0.20	0.60	0.27	0.16	0.47

All statistical tests were conducted with α = 0.05

## Discussion

In this study of US pregnant and postpartum individuals resulting from an online survey, between a quarter and a third of participants expressed a willingness to participate in clinical trials for medications or vaccines in pregnancy or while breastfeeding. Those with greater educational attainment, who lived in urban settings, and had chronic medical conditions were more likely to be willing to participate. Personal medical benefit and altruism were common motivators of participation and safety concerns for both oneself and the baby or fetus were nearly ubiquitous deterrents of participation. Those with altruistic motivations were most likely to be willing to participate in trials while pregnant or breastfeeding, and personal medical benefit served to motivate likelihood of participation in pregnancy only. Safety concerns, discomfort with experimentation, and distrust in pharmaceutical companies were factors most consistently associated with a reduced willingness to participate in trials. This work provides an in-depth examination of the perspectives of pregnant and lactating individuals with regard to their attitudes and preferences surrounding clinical trial participation, thus providing foundational information for clinical trial sponsors and regulatory authorities that can be used to meet the needs of this critical population.

Results from this study are consistent with data from other qualitative studies evaluating perspectives of participation in clinical trials in pregnant and lactating individuals. In a study among pregnant people enrolled in an observational trial, the main motivators of participation were a willingness to help medical research and having a personal connection to the disease [30]. While it has consistently been found that altruism and potential health benefits for oneself and the fetus/baby were key motivators, without the tangible support required to accommodate the unique needs of these populations, these motivators may not be enough to increase or sustain recruitment. Offering substantial incentives such as childcare and active recruitment strategies involving a prenatal care team have shown to improve recruitment and retention [31, 32]. A recently published systematic review of studies documenting pregnant peoples’ reasons for participation in clinical research demonstrated that altruistic motives were the most common, ranging from desires to help other pregnant women and babies as well as advancing science [33]. Our study echoed these same sentiments, with items in the altruism domain being cited as important motivators among more than 60% of participants, especially among those with chronic medical conditions (74.8% and 66.5% in pregnancy and lactation, respectively).

Another main finding similar to other studies was the nearly unanimous concern about the safety of the fetus or baby as a deterrent for participation in clinical trials. Despite this, about a third of respondents reported that they would be likely to participate in a clinical trial during pregnancy or lactation. The topic of a medication’s safety profile prior to initiating a trial in pregnant or lactating people is critical and often complex. Due to the nature of trials for investigational products, offering full clinical safety information prior to enrollment will likely not be possible. In a study evaluating the perception of risks and benefits of participation in vaccine trials in pregnancy, women expressed high value on evidence in general, and pointed to it as a primary motivator to accept versus decline participation [19]. This study’s results suggest that different types and amounts of data may importantly influence people’s views about clinical trial participation during pregnancy. Pregnant people seek information beyond animal data due to concerns about the lack of specificity linking required animal studies to human reproductive risk [34]. In addition, pregnant people have varying degrees of acceptance of the clinical evidence in non-pregnant people as relevant to informing their decision [19, 35]. Not only does the potential for fetal exposure differentiate pregnant and non-pregnant study participants, but pregnancy induces physiological changes that alter drug absorption, distribution, metabolism, and excretion, potentially impacting drug safety and efficacy [36]. Beyond full disclosure of the available evidence at the time of consent for participating in a clinical trial, a full assessment weighing the relative risks and benefits of the intervention versus the alternatives by the clinician investigator with the pregnant person can help build trust and promote participation. At a minimum, our survey results underscore the need for sponsors to explain the level of monitoring that would occur for both the fetus/baby and mother in such a trial to assuage this ubiquitous safety concern.

In addition to motivators and deterrents, we examined the potential impact of educational attainment and household income on willingness to participate in trials as well as motivator and deterrent endorsement, as a proxy for socioeconomic status. Clear patterns emerged specifically with educational attainment: those who were more educated were more likely to report being willing to participate in trials for both medications and vaccines, both while pregnant and breastfeeding. Further, more educated individuals were less likely to be motivated by financial compensation and deterred by logistical challenges, dislike of medical interventions, distrust in pharmaceutical companies, and being uncomfortable with experimentation, which may highlight the privilege of those with greater socioeconomic position. In order to recruit diverse participants for clinical trials overall, as well as in pregnancy and lactation, targeted education and outreach efforts are needed to make access to information and resources more equitable [37]. Further, recruiting clinical investigators from diverse backgrounds that reflect the community can increase the level of trust and willingness to enroll participants that reflect a wider range of socioeconomic, racial, ethnic, and gender minority groups [38].

Our study had several strengths and limitations. First, this study was comprehensive in its coverage of different types of clinical trials (i.e., for new medications and vaccines), which represents a broad range of situations that likely have unique motivators and deterrents for participation. For example, participating in a trial for an experimental medication to treat a specific condition is different than a trial for a new vaccine to prevent an infection or disease. In addition, our study was unique in its assessment of both pregnant and lactating people, the latter of which are infrequently studied. Second, our analysis of motivators and deterrents highlighted the consideration of both the risks and benefits that pregnant and lactating individuals have to weigh in deciding to participate in a clinical trial. However, we did not conduct a ranked choice experiment, where participants could rank motivators and deterrents in order of importance. This type of design could have elucidated whether certain motivators drove the decision or just supported it. For example, while we observed that altruism was a significant predictor of participation in both medication and vaccine trials across both pregnancy and lactation, it is not clear from the data whether altruism alone was the reason pregnant or lactating people would participate in trials. Social desirability response bias, functioning as a tendency for participants to overreport socially accepted attitudes or behaviors, also could have played a role in these findings related to altruism [39]. Finally, though we asked about a wide array of factors that may influence research participation, participants provided additional considerations in response to our open-ended question at the end of the survey. These included current management of disease with standard of care therapies; benefit-risk balance, especially if the baby had a high-risk condition; the stage of development for the investigational product; mechanism of action for the investigational product and if it was similar to standard of care; and the financial contributors of the study. Future studies may take these nuanced and complex concepts into account.

While we documented a substantial likelihood of participation, we note that the participants in this study do not represent a random sample of all pregnant or lactating people. Of note, 15% reported that they had participated in a trial in the past, which suggests that this population is more comfortable with trials than the general population, of which less than 5% have participated in a clinical trial [40, 41]. This may have occurred because of our targeted advertisements that likely garnered more interest from individuals with this inclination in addition to the notion that those who voluntarily participate in a survey are more likely to be willing to participate in research than those who decline. In addition, using social media platforms for recruitment likely contributed to the non-representativeness of our study sample [42]. Specifically, our study sample was demographically homogenous, with 84% being white and 91% non-Hispanic. Therefore, we were unable to examine whether determinants of clinical trial participation in pregnancy and lactation varied by race or ethnicity. We urge future researchers to examine factors influencing participation among racial minorities [43], as there is a pressing need to increase inclusion among these groups in clinical trials [44]. Furthermore, our study was only conducted online in the US and in English, so findings may not generalize to other countries or settings. Finally, interrater reliability in the interpretation of survey questions was not assessed, most survey instruments were not validated, and all data were self-reported.

Despite these limitations, our study provides novel insights from a comprehensive assessment of the perspectives, attitudes, and dispositions regarding participation in clinical trials for both experimental medications and vaccines during pregnancy and lactation. With the timing of this survey, we were able to capitalize on the post-COVID-19 pandemic perspective, as the pandemic elevated the importance of this topic, especially among pregnant and lactating populations. This study lays the foundation for future research in more diverse settings.

## Conclusions

While policy-level initiatives to include pregnant and lactating people in clinical trials are gaining momentum, accommodations in the design and conduct of clinical trials are lagging. This study provides key data to inform inclusive recruitment strategies aimed at achieving equity in evidence generation for pregnant and lactating people. Despite frequently cited motivators for participation including personal medical benefit and altruism, safety concerns specifically for the baby or fetus were pervasive. While the reported willingness to participate in trials was substantial in this study, these findings highlight the unique and nuanced perspectives of pregnant and postpartum individuals that must be addressed in order to meaningfully increase participation.

### Electronic supplementary material

Below is the link to the electronic supplementary material.

Supplementary Material 1

Supplementary Material 2

Supplementary Material 3

## Data Availability

Data can be accessed by contacting study authors.
