# Supplementary figures and images for: Understanding willingness and barriers to participate in clinical trials during pregnancy and lactation: findings from a US study
Source: BMC Pregnancy Childbirth. 2024 Jul 26;24:504. doi: 10.1186/s12884-024-06710-w (PMC11282851; doi:10.1186/s12884-024-06710-w)

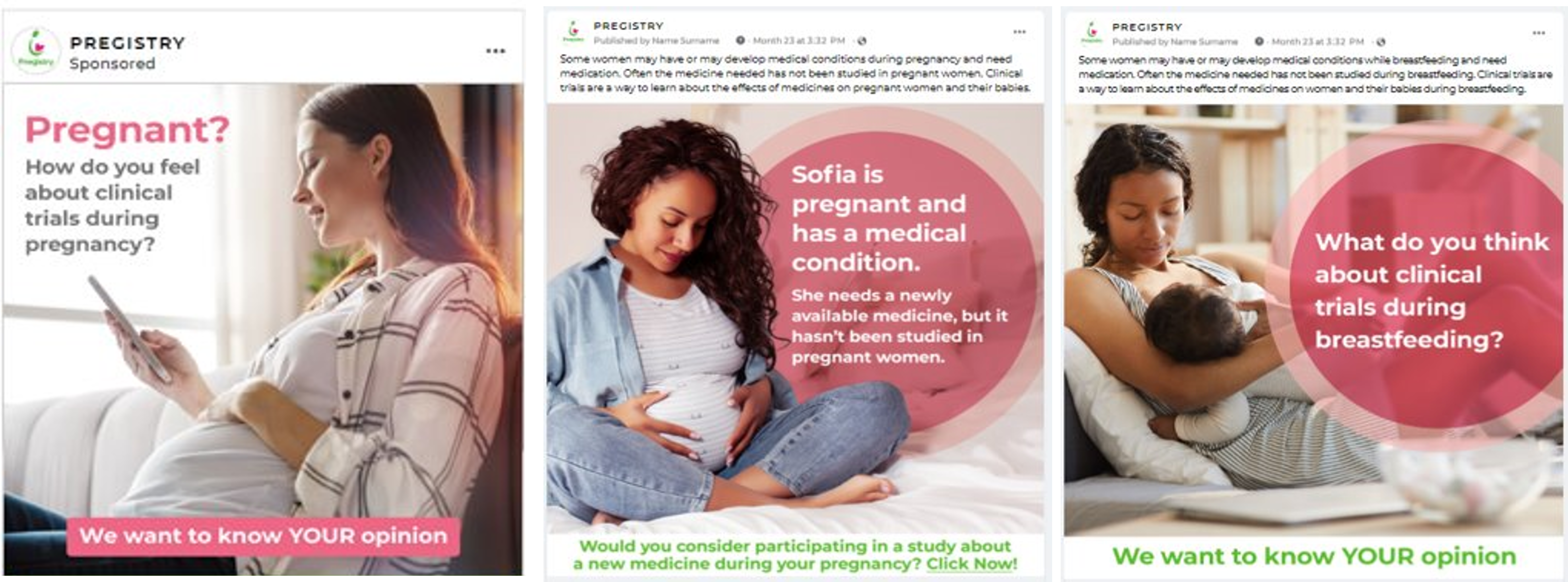

Supplement: Supplementary file 1 — Supplementary Material 1 [file 12884_2024_6710_MOESM1_ESM.png]
