# Supplementary material for: Understanding willingness and barriers to participate in clinical trials during pregnancy and lactation: findings from a US study
Source: BMC Pregnancy Childbirth. 2024 Jul 26;24:504. doi: 10.1186/s12884-024-06710-w (PMC11282851; doi:10.1186/s12884-024-06710-w)
Supplement: Supplementary file 2 — Supplementary Material 2 [file 12884_2024_6710_MOESM2_ESM.docx]

**Supplemental Material to Jacobson et al., Understanding Willingness and Barriers to Participate in Clinical Trials during Pregnancy and Lactation: Findings from a US Study**

**Survey Instrument**

**INTRODUCTION**

Your opinion is valuable to us.

We appreciate you taking the time to complete the following anonymous questionnaire. It should take approximately 10 minutes to complete. The answers you provide will enable us to better understand your perspectives regarding participation in clinical trials during pregnancy and/or lactation.

This questionnaire does not intend to capture any drug adverse event or safety complaints.

Thank you for your participation.

**SECTION 1: DEMOGRAPHICS**

1. **Do you live in the United States?**

• Yes
• No [NOT A CANDIDATE] → Message: “Sorry, you are not eligible to participate.”

1. **In which state do you live?**

(Dropdown with US states)

1. **Please indicate how urban your living environment is:**

Rural

(houses are very far apart)

Urban

(living in cities)

Suburban

(mix of urban and rural)

**4. What is your current age?** *(Persons younger than 18 years or older than 50 years are not eligible* → Message: “Sorry, you are not eligible to participate.” *)*

(Dropdown list from 18 to 50)

**5. Are you currently pregnant?**
• Yes *Go to question 6*
• No *Go to question 8*

**6. How many weeks pregnant are you approximately?**

- Less than 12 weeks
- 12 – 16 weeks
- 17 – 20 weeks
- 21 – 30 weeks
- 31 – 36 weeks
- 37 or more weeks

**7. Are you pregnant with more than one baby?**
• Yes *Go to question 16*
• No *Go to question 16*

• I don’t know *Go to question 16*

**8. Have you been pregnant during the past 12 months?**

• Yes
• No [NOT A CANDIDATE] → Message: “Sorry, you are not eligible to participate”

**9. Did you have a baby (live birth) during the past 12 months?**

• Yes *Go to question 11*
• No *Go to question 10*

**10.** **What was the outcome of your pregnancy?**

- I had a miscarriage / spontaneous abortion (before 20 weeks gestation) *Go to question 16*
- I had a stillbirth (at or after 20 weeks gestation) *Go to question 16*
- I had an ectopic / tubal pregnancy *Go to question 16*
- Other *Go to question 16*
- I prefer to not specify  *Go to question 16*

**11. How old is your baby?**

- Less than 3 months
- Between 3 months and less than 6 months
- Between 6 months and less than 9 months
- Between 9 months and less than one year

**12. Are you currently breastfeeding or pumping breastmilk to feed your baby?**

• Yes *Go to Question 15*

• No *Go to Question 13*

**13. Did you breastfeed or pump breastmilk for your baby *for longer than 2 weeks* at any time after delivery?**

• Yes

• No *Go to Question 15*

**14. How long did you breastfeed or pump breastmilk for your baby?**

- Between 2 weeks and less than 1 month
- Between 1 month and less than 3 months
- Between 3 months and less than 6 months
- Between 6 months and less than one year

**15. What kind(s) of milk are you feeding your baby now? (Select all that apply)**

- My breastmilk only
- Formula feeding only
- My breastmilk and formula
- Other (e.g., cow’s milk)

**16. How many times have you previously been pregnant? (*For those currently pregnant:* Not including your current pregnancy)**

(Numeric values: 0 to 20)

**17. How many past pregnancies ended in a live birth?**

(Numeric values: 0 to 20)

**18. How many past pregnancies did not end in a live birth (e.g., stillbirth, miscarriage / spontaneous abortion, ectopic / tubal pregnancy, other)?**

(Numeric values: 0 to 20)

**19. Which race(s) do you most identify with? (Select all that apply)**

- White
- Black or African American
- American Indian or Alaska Native
- Asian
- Native Hawaiian or Other Pacific Islander
- Other

**20. Which ethnicity do you most identify with?**

- Latinx / Hispanic
- Not Latinx / Hispanic

**21. What is your marital status?**• Single
• Married and living with my partner

• Separated
• Unmarried and living with my partner

• Divorced

• Widowed

**22. What is the highest level of education you completed?**• Never attended school
• Elementary school
• Some high school
• High school graduate or general equivalency diploma (GED)

- Trade school

• Some college/university
• Bachelor's degree (e.g., BA, BS) 
• Master's degree (e.g., MA, MS, MEng, MEd, MSW, MBA)

- Professional degree (e.g., MD, DDS, DVM, JD, PhD)

**23. Which of the following best describes your employment status at the time you became pregnant?**

- Employed full time (35 or more hours per week)
- Employed part time (less than 35 hours per week)
- Employed but on maternity leave
- Not employed, looking for work
- Not employed, not looking for work
- Disabled, not able to work

**24. Which of the following best describes your total yearly household income before taxes or other deductions at the time you became pregnant?**

- Less than $10,000
- Between $10,00 and less than $40,000
- Between $40,000 and less than $90,000
- Between 90,000 and less than $150,000
- Between $150,000 and less than $250,000
- $250,000 or more
- I don’t know
- I prefer not to answer

**25. Do you work in, or have you ever worked in a healthcare or clinical research field?**

• Yes
• No

**26. Do you have medical insurance through the government, your employer, yourself, or a family member?**
• Yes *Go to Question 27*
• No *Go to Question 28*

**27. What type of insurance do you have? (please select all that apply)**

- Public (e.g., from the government [Medicaid])
- Private

**SECTION 2: HEALTH**

**28. How was your general health when you became pregnant?**

- Excellent (totally healthy, no illness)
- Very good
- Good (some health issues, but generally well)
- Fair
- Poor (significant health problems)

**29. Please select which medical conditions, if any, you had at the time you became pregnant (Select all that apply).**

- None
- Arrhythmia
- Asthma
- Cancer
- Chronic liver disease
- Chronic renal disease
- Crohn's disease
- Deep vein thrombosis
- Diabetes
- Endometriosis
- Epilepsy
- Heart failure
- Hepatitis
- High blood pressure
- HIV / AIDS
- Inflammatory bowel disease
- Low blood pressure
- Lupus
- Mental health condition (e.g., anxiety, depression, etc. )
- Migraine
- Multiple sclerosis
- Polycystic ovary syndrome (PCOS)
- Rheumatoid arthritis
- Sarcoidosis
- Sjogren’s syndrome
- Systemic lupus erythematosus
- Thyroid condition
- Other

[For any response to above (medical conditions) except None]

**30. Are you currently taking medication(s) for your medical condition(s) as indicated above?**

- Yes
- No

**31. Have you been vaccinated for COVID-19?**

- Yes *Go to question 32*
- No *Go to question 33 then 36 [currently pregnant] or 34, 35, 36 [postpartum – live birth] or 36 [postpartum - not live birth]*
- I do not wish to answer *Go to question 33 or 34 or 36 per the directions for “No”*

**32. Did you get vaccinated for COVID-19 during pregnancy or while breastfeeding?**

- Yes, I was pregnant
- Yes, I was breastfeeding
- Yes, I was pregnant and breastfeeding
- No, I was not pregnant nor breastfeeding
- I do not wish to answer

**FOR CURRENTLY PREGNANT - YES**

**33. Do you plan to have your child/children receive routine childhood immunizations according to the recommended vaccination schedule?**

- Yes *Go to question 36*
- No *Go to question 36*
- Not sure *Go to question 36*

**FOR POST PARTUM– LIVE BIRTH**

**34. Have you had your child/children receive routine childhood immunizations according to the recommended vaccination schedule?**

- Yes
- No
- Not sure
- My child/children aren’t old enough for any routine vaccinations yet

**FOR POSTPARTUM - LIVE BIRTH**

**35. Does/did your baby have any serious medical problems or birth defects?**

- Yes
- No
- Not sure

**SECTION 3: CLINICAL TRIALS**

This section contains question about **clinical trials**. **Clinical trials** are research studies performed in people (volunteers) that test and evaluate new medicines, new vaccines, or new medical devices. They are the primary way that researchers find out if a new treatment is safe and effective in people (adapted from <https://www.nia.nih.gov/health/what-are-clinical-trials-and-studies>)]

***Where needed, the question may pertain to only certain subjects, such as only those currently pregnant (Question 5); only those who are postpartum and had a live birth (Question 9) and breastfed/are breastfeeding (Questions 12 and 13); and those with chronic medical conditions (Question 29). If not specified, then the question is for ALL participants*.**

**36. When a new medicine becomes available, how confident are you that the medicine will be safe and with no serious (harmful) side effects?**

- Very confident
- Fairly confident
- Somewhat confident
- Not very confident
- Not at all confident

**37. When a new medicine becomes available, how confident are you that the medicine will work?**

- Very confident
- Fairly confident
- Somewhat confident
- Not very confident
- Not at all confident

**38. How familiar are you with clinical trials?**

- I have a good understanding about clinical trials
- I know some facts about clinical trials
- I have heard of clinical trials, but don’t really know what they are
- I have never heard of clinical trials

**39. Have you ever participated in a clinical trial?**

- Yes *Go to question 40*
- No *Go to question 41*
- I don’t know *Go to question 41*

**40. Were you pregnant or breastfeeding at the time you participated in the clinical trial?**

- I was pregnant
- I was breastfeeding
- I was pregnant and breastfeeding
- I was neither pregnant nor breastfeeding
- I am not sure if I was pregnant or breastfeeding

**41. Have you ever discussed participating in a clinical trial with your health care provider?**

- Yes
- No

**42. Please rate how much you agree with the following statement: "I believe clinical trials should be conducted for new medicines, vaccines, or medical devices."**

- Strongly agree
- Agree
- No opinion
- Disagree
- Strongly disagree

***SELECTS a MEDICAL CONDITION: USE THIS QUESTION***

**43. Given you have a medical condition, how willing would you be to participate in a clinical trial during pregnancy for a new medicine to treat this medical condition?**

- Extremely likely
- Likely
- Neutral/not sure
- Unlikely
- Extremely unlikely

***SELECTS “NONE” for MEDICAL CONDITION: USE THIS QUESTION***

**44. Suppose you have a medical condition (such as asthma, lupus, or Crohn’s disease). How willing would you be to participate in a clinical trial during your pregnancy for a new medicine to treat the medical condition?**

- Extremely likely
- Likely
- Neutral/not sure
- Unlikely
- Extremely unlikely

**45. Would you be willing to participate in a clinical trial for a new vaccine during pregnancy?**

- Extremely likely
- Likely
- Neutral/not sure
- Unlikely
- Extremely unlikely

**FOR CURRENTLY PREGNANT - YES**

**46. How would your opinion about clinical trials change if you were not pregnant?**

- I would be more likely to participate
- My opinion would not change
- I would be less likely to participate

**47. When thinking about participating in a clinical trial during pregnancy, would the trimester of your pregnancy influence your willingness to participate?**

- Yes, I would be more likely to participate during my 2^nd^ or 3^rd^ trimester than my 1^st^ trimester
- Yes, I would be more likely to participate during my 1st trimester than my 2^nd^ or 3^rd^ trimester
- No, I would be equally likely to participate (or not participate) during any trimester

**FOR POST PARTUM– LIVE BIRTH and BREASTFED/BREASTFEEDING**

***SELECTS a MEDICAL CONDITION: USE THIS QUESTION***

**48. Given you have a medical condition, how willing would you be to participate in a clinical trial while breastfeeding your baby for a new medicine to treat this medical condition?**

- Extremely likely
- Likely
- Neutral/not sure
- Unlikely
- Extremely unlikely

**FOR POST PARTUM– LIVE BIRTH and BREASTFED/BREASTFEEDING**

***SELECTS “NONE” for MEDICAL CONDITION: USE THIS QUESTION***

**49. Suppose you have a medical condition (such as asthma, lupus, or Crohn’s disease). How willing would you be to participate in a clinical trial while breastfeeding for a new medicine to treat this medical condition?**

- Extremely likely
- Likely
- Neutral/not sure
- Unlikely
- Extremely unlikely

**FOR POST PARTUM– LIVE BIRTH and BREASTFED/BREASTFEEDING**

**50. Would you be willing to participate in a clinical trial for a new vaccine while breastfeeding your baby?**

- Extremely likely
- Likely
- Neutral/not sure
- Unlikely
- Extremely unlikely

**FOR POST PARTUM– LIVE BIRTH and BREASTFED/BREASTFEEDING**

**51. How would your opinion about clinical trials change if you were not (or did not) breastfeeding your baby?**

- I would be more likely to participate
- My opinion would not change
- I would be less likely to participate

**52. Has your opinion on clinical trials changed after the COVID-19 pandemic?**

- Yes - I think they are more important now
- Yes - I think they are less important now
- No - My opinion has not changed
- Maybe
- I don’t know

**53.** **What information would you consider to be important when deciding whether to participate in a clinical trial during pregnancy?**

**Each of the following items will have a Likert scale for response selection**

Not at all important

Slightly important

Extremely important

Moderately important

Very important

- What medical condition the new medicine (or new vaccine) is going to treat
- If the medical condition being studied in the clinical trial worsens during pregnancy
- If any side effect(s) occurred in animals that were exposed to the new medicine (or new vaccine)
- How effective the new medicine (or new vaccine) is in nonpregnant people
- If any side effect(s) occurred in nonpregnant people that were exposed to the new medicine (or new vaccine)
- How many pregnant women have previously received the new medicine (or new vaccine) in clinical trials
- Likelihood of side effects from the new medicine (or new vaccine) for my developing baby
- If the information / explanations will be in my native language and easy to understand
- How much money I would be paid for my participation

**FOR POST PARTUM– LIVE BIRTH and BREASTFED/BREASTFEEDING**

**54. What information would you consider to be important when deciding whether to participate in a clinical trial while breastfeeding your baby?**

**Each of the following items will have a Likert scale for response selection**

Not at all important

Slightly important

Extremely important

Moderately important

Very important

- What medical condition the new medicine (or new vaccine) is going to treat
- If any side effect(s) occurred in animals that were exposed to the new medicine (or new vaccine) during testing
- If the new medicine (or new vaccine) was detected in the breastmilk of exposed and lactating animals
- How effective the new medicine (or new vaccine) is in nonpregnant people
- If any side effect(s) occurred in nonpregnant people that were exposed to the new medicine (or new vaccine)
- Likelihood of side effects from the new medicine (or new vaccine) for my baby
- If the information / explanations will be in my native language and easy to understand
- How much I would be compensated for my participation

**55. Please rate how important each of the following below would be to ENCOURAGE you to participate in a clinical trial during pregnancy? (ALL can answer this question)**

**Each of the following items will have a Likert scale for response selection**

Not at all important

Slightly important

Extremely important

Moderately important

Very important

INCLUDE the items below with an asterisk (*) only for participants that ***SELECTED a MEDICAL CONDITION***

- To help other people with the same medical problem*
- To contribute to (to advance) scientific / medical research
- Out of gratitude to the medical / research community
- To help researchers find new / better treatments
- Severity of my medical condition*
- To obtain information about my disease*
- To have a more active role in my healthcare
- To improve my own medical treatment*
- Potential benefit to my health
- To receive extra medical attention and monitoring during my pregnancy
- Financial compensation
- Because my doctor or healthcare provider recommended it
- Encouraged to or persuaded by family / friends / other trusted individuals
- Encouraged to or persuaded by my partner
- Curiosity
- To receive treatment for my medical condition not available outside of the clinical trial*
- To receive the findings of the clinical trial
- To pass the time
- Because I am against animal experiments

**FOR POST PARTUM– LIVE BIRTH and BREASTFED/BREASTFEEDING**

**56. Please rate how important each of the following below would be to ENCOURAGE you to participate in a clinical trial while breastfeeding your baby?**

**Each of the following items will have a Likert scale for response selection**

Not at all important

Slightly important

Extremely important

Moderately important

Very important

INCLUDE the items below with an asterisk (*) only for participants that ***SELECTED a MEDICAL CONDITION***

- To help other people with the same medical problem*
- To contribute to (to advance) scientific / medical research
- Out of gratitude to the medical / research community
- To help researchers find new / better treatments
- Severity of my medical condition*
- To obtain information about my disease*
- To have a more active role in my healthcare
- To improve my own medical treatment*
- Potential benefit to my health
- To receive extra medical attention and monitoring
- Financial compensation
- Because my doctor or healthcare provider recommended it
- Encouraged to or persuaded by family / friends / other trusted individuals
- Encouraged to or persuaded by my partner
- Curiosity
- To receive treatment for my medical condition not available outside of the clinical trial*
- To receive the findings of the clinical trial
- To pass the time
- Because I am against animal experiments

**57. Please rate how important each of the following below would be to DISCOURAGE you from participating in a clinical trial during pregnancy? (ALL can answer this question)**

**Each of the following items will have a Likert scale for response selection**

Not at all important

Slightly important

Extremely important

Moderately important

Very important

- Possible side effects on me
- Possible side effects on my developing baby
- Possible risk to my future fertility
- Possible risk to the pregnancy (e.g., miscarriage)
- No perceived benefit to me or to my baby
- I would worry about long-term unknown effects on the health of my baby
- Lack of trust in researchers / pharmaceutical companies
- I had a prior pregnancy complication
- Time commitment – the clinical trial will take too much time
- I am concerned that the clinical trial visits or participation would conflict with my work
- I do not have reliable transportation
- I do not have childcare for my child/children so that I can attend clinical trial appointments
- Not acceptable by my family / friends / other trusted individuals
- Not acceptable by my partner
- Don’t want to be experimented upon
- Don’t like hospitals or doctor’s offices
- I am concerned about having my blood drawn
- I am concerned about undergoing medical tests / X-rays
- I am concerned that a procedure may cause distress or pain to my developing baby

**FOR POST PARTUM– LIVE BIRTH and BREASTFED/BREASTFEEDING**

**58. Please rate how important each of the following below would be to DISCOURAGE you from participating in a clinical trial while breastfeeding?**

**Each of the following items will have a Likert scale for response selection**

Not at all important

Slightly important

Extremely important

Moderately important

Very important

- Possible side effects on me
- Possible side effects on my baby
- Possible effect on my milk production
- Concern about interruption of breastfeeding
- Possible risk to my future fertility
- No perceived benefit to me or to my baby
- I would worry about long-term unknown effects on the health of my baby
- Lack of trust in researchers / pharmaceutical companies
- Time commitment – the clinical trial will take too much time
- I am concerned that the clinical trial visits or participation would conflict with my work
- I do not have reliable transportation
- I do not have childcare for my child/children so that I can attend clinical trial appointments
- Not acceptable by my family / friends / other trusted individuals
- Not acceptable by my partner
- Don’t want to be experimented upon
- Don’t like hospitals or doctor’s offices
- I am concerned about having my blood drawn
- I am concerned about undergoing medical tests / X-rays

**One type of clinical trial is a “Randomized Controlled Trial” where there are two groups of people who are treated exactly the same, except only one group gets the true medicine under investigation. The other group gets a ‘placebo’ (inactive) product.**

**The volunteer does not get to choose whether they receive the true medicine or placebo.**

**59. Suppose you have a medical condition. *After reading the above information,*** **would you be willing to participate in a randomized clinical trial during pregnancy for a new medicine to treat this medical condition?**

- I would be less likely to take part as I would want to guarantee that I would have the true medicine
- I would be more likely to take part as I might not get the true medicine
- This would not affect my decision

**FOR POST PARTUM– LIVE BIRTH and BREASTFED/BREASTFEEDING**

***60. After reading the above information,*** **would you be willing to participate in a randomized clinical for a new medicine while breastfeeding your baby?**

- I would be less likely to take part as I would want to guarantee that I would have the true medicine
- I would be more likely to take part as I might not get the true medicine
- This would not affect my decision

**61. Is there anything else we did not ask about that would influence your decision to participate in a clinical trial?**

(Text Field)

**Thank you very much for completing this survey!**

The success of this study depends on the voluntary participation of pregnant and recently pregnant women across the US!

**FEEDBACK**

**We would like to receive your feedback about this survey:**

(Text Field)

**How did you hear about us? (Select all that apply)**

- Google
- Facebook ad
- A website
- A post from a friend on social media
- Word of mouth
- Other
