# Supplementary material for: Understanding willingness and barriers to participate in clinical trials during pregnancy and lactation: findings from a US study
Source: BMC Pregnancy Childbirth. 2024 Jul 26;24:504. doi: 10.1186/s12884-024-06710-w (PMC11282851; doi:10.1186/s12884-024-06710-w)
Supplement: Supplementary file 3 — Supplementary Material 3 [file 12884_2024_6710_MOESM3_ESM.docx]

| **Supplemental Table 1**. Participant characteristics by likelihood of participation in a clinical trial for a medication or vaccine while breastfeeding (n=504) | | | | | | | | | | | | |
| --- | --- | --- | --- | --- | --- | --- | --- | --- | --- | --- | --- | --- |
|  | Overall | | Participation in a clinical trial for a medication | | | |  | Participation in a clinical trial for a vaccine | | | |  |
|  |  |  | Likely (206, 41%) | | Not likely (n=298, 59%) | |  | Likely (n=210, 42%) | | Not likely (n=294, 58%) | |  |
| Characteristic | N | % | N | % | N | % | p | N | % | N | % | p |
| Pregnancy status |  |  |  |  |  |  | - |  |  |  |  | - |
| Pregnant | 0 | 0.0 | 0 | 0.0 | 0 | 0.0 |  | 0 | 0.0 | 0 | 0.0 |  |
| Postpartum | 504 | 100.0 | 206 | 100 | 298 | 100 |  | 210 | 100 | 294 | 100 |  |
| Breastfeeding behavior (among postpartum only) |  |  |  |  |  |  | - |  |  |  |  | - |
| Ever breastfed | 504 | 100.0 | 206 | 100 | 298 | 100 |  | 210 | 100 | 294 | 100 |  |
| Never breastfed | 0 | 0.0 | 0 | 0.0 | 0 | 0.0 |  | 0 | 0.0 | 0 | 0.0 |  |
| Age (years) |  |  |  |  |  |  | 0.23 |  |  |  |  | 0.56 |
| 18-24 | 55 | 10.9 | 17 | 8.3 | 38 | 12.8 |  | 18 | 8.6 | 37 | 12.6 |  |
| 25-29 | 115 | 22.8 | 54 | 26.2 | 61 | 20.5 |  | 50 | 23.8 | 65 | 22.1 |  |
| 30-34 | 187 | 37.1 | 78 | 37.9 | 109 | 36.6 |  | 79 | 37.6 | 108 | 36.7 |  |
| 35-44 | 147 | 29.2 | 57 | 27.7 | 90 | 30.2 |  | 63 | 30.0 | 84 | 28.6 |  |
| Mean (SD) | 31.6 | 5.1 | 31.5 | 4.8 | 31.6 | 5.3 | 0.85 | 31.7 | 5.0 | 31.5 | 5.2 | 0.60 |
| Race |  |  |  |  |  |  | 0.26 |  |  |  |  | 0.08 |
| White | 429 | 85.5 | 175 | 85.4 | 254 | 85.5 |  | 177 | 84.7 | 252 | 86.0 |  |
| Black | 16 | 3.2 | 8 | 3.9 | 8 | 2.7 |  | 9 | 4.3 | 7 | 2.4 |  |
| American Indian/Alaska Native | 3 | 0.6 | 3 | 1.5 | 0 | 0.0 |  | 3 | 1.4 | 0 | 0.0 |  |
| Asian | 14 | 2.8 | 7 | 3.4 | 7 | 2.4 |  | 8 | 3.8 | 6 | 2.1 |  |
| Pacific Islander | 1 | 0.2 | 0 | 0.0 | 1 | 0.3 |  | 0 | 0.0 | 1 | 0.3 |  |
| Other | 16 | 3.2 | 5 | 2.4 | 11 | 3.7 |  | 3 | 1.4 | 13 | 4.4 |  |
| Multiracial | 23 | 4.6 | 7 | 3.4 | 16 | 5.4 |  | 9 | 4.3 | 14 | 4.8 |  |
| Ethnicity |  |  |  |  |  |  | 0.88 |  |  |  |  | 0.50 |
| Non-Hispanic | 449 | 91.3 | 183 | 91.5 | 266 | 91.1 |  | 185 | 90.2 | 264 | 92.0 |  |
| Hispanic | 43 | 8.7 | 17 | 8.5 | 26 | 8.9 |  | 20 | 9.8 | 23 | 8.0 |  |
| Education |  |  |  |  |  |  | 0.20 |  |  |  |  | <0.01 |
| ≤High school | 68 | 13.5 | 26 | 12.6 | 42 | 14.1 |  | 21 | 10.0 | 47 | 16.0 |  |
| Some college or trade school | 182 | 36.1 | 65 | 31.6 | 117 | 39.3 |  | 64 | 30.5 | 118 | 40.1 |  |
| Bachelor's degree | 140 | 27.8 | 61 | 29.6 | 79 | 26.5 |  | 59 | 28.1 | 81 | 27.6 |  |
| Postgraduate | 114 | 22.6 | 54 | 26.2 | 60 | 20.1 |  | 66 | 31.4 | 48 | 16.3 |  |
| Household income |  |  |  |  |  |  | 0.17 |  |  |  |  | 0.26 |
| Prefer not to answer or don't know | 25 |  | 6 |  | 19 |  |  | 4 |  | 21 |  |  |
| <$10,000-<$40,000 | 110 | 23.0 | 42 | 21.0 | 68 | 24.4 |  | 44 | 21.4 | 66 | 24.2 |  |
| ≥$40,000-<$90,000 | 190 | 39.7 | 85 | 42.5 | 105 | 37.6 |  | 77 | 37.4 | 113 | 41.4 |  |
| ≥$90,000-<$150,000 | 120 | 25.1 | 43 | 21.5 | 77 | 27.6 |  | 53 | 25.7 | 67 | 24.5 |  |
| ≥$150,000 | 59 | 12.3 | 30 | 15.0 | 29 | 10.4 |  | 32 | 15.5 | 27 | 9.9 |  |
| Urbanicity |  |  |  |  |  |  | 0.06 |  |  |  |  | 0.03 |
| Rural | 82 | 16.3 | 28 | 13.6 | 54 | 18.1 |  | 25 | 11.9 | 57 | 19.4 |  |
| Suburban | 334 | 66.3 | 133 | 64.6 | 201 | 67.5 |  | 141 | 67.1 | 193 | 65.7 |  |
| Urban | 88 | 17.5 | 45 | 21.8 | 43 | 14.4 |  | 44 | 21.0 | 44 | 15.0 |  |
| Marital status |  |  |  |  |  |  | 0.03 |  |  |  |  | 0.60 |
| Married or cohabitating | 476 | 94.4 | 189 | 91.8 | 287 | 96.3 |  | 197 | 93.8 | 279 | 94.9 |  |
| Single | 28 | 5.6 | 17 | 8.3 | 11 | 3.7 |  | 13 | 6.2 | 15 | 5.1 |  |
| Chronic medical condition |  |  |  |  |  |  | 0.08 |  |  |  |  | 0.03 |
| No | 194 | 38.5 | 70 | 34.0 | 124 | 41.6 |  | 69 | 32.9 | 125 | 42.5 |  |
| Yes | 310 | 61.5 | 136 | 66.0 | 174 | 58.4 |  | 141 | 67.1 | 169 | 57.5 |  |
| Gravidity |  |  |  |  |  |  | 0.29 |  |  |  |  | 0.20 |
| Primigravida | 173 | 34.6 | 65 | 31.9 | 108 | 36.5 |  | 79 | 37.8 | 94 | 32.3 |  |
| Multigravida | 327 | 65.4 | 139 | 68.1 | 188 | 63.5 |  | 130 | 62.2 | 197 | 67.7 |  |
| History of non-live birth |  |  |  |  |  |  | 0.82 |  |  |  |  | 0.86 |
| No | 280 | 56.0 | 113 | 55.4 | 167 | 56.4 |  | 118 | 56.5 | 162 | 55.7 |  |
| Yes | 220 | 44.0 | 91 | 44.6 | 129 | 43.6 |  | 91 | 43.5 | 129 | 44.3 |  |
| Worked in healthcare |  |  |  |  |  |  | 0.26 |  |  |  |  | 0.97 |
| No | 310 | 61.6 | 133 | 64.6 | 177 | 59.6 |  | 129 | 61.7 | 181 | 61.6 |  |
| Yes | 193 | 38.4 | 73 | 35.4 | 120 | 40.4 |  | 80 | 38.3 | 113 | 38.4 |  |
| Prior participation in clinical trial |  |  |  |  |  |  | 0.02 |  |  |  |  | <0.01 |
| No | 434 | 87.7 | 169 | 83.7 | 265 | 90.4 |  | 169 | 82.0 | 265 | 91.7 |  |
| Yes | 61 | 12.3 | 33 | 16.3 | 28 | 9.6 |  | 37 | 18.0 | 24 | 8.3 |  |
| General health |  |  |  |  |  |  | 0.42 |  |  |  |  | 0.07 |
| Poor or fair | 22 | 4.4 | 12 | 5.9 | 10 | 3.4 |  | 15 | 7.1 | 7 | 2.4 |  |
| Good | 160 | 31.8 | 67 | 32.7 | 93 | 31.2 |  | 63 | 30.0 | 97 | 33.1 |  |
| Very Good | 175 | 34.8 | 65 | 31.7 | 110 | 36.9 |  | 75 | 35.7 | 100 | 34.1 |  |
| Excellent | 146 | 29.0 | 61 | 29.8 | 85 | 28.5 |  | 57 | 27.1 | 89 | 30.4 |  |

All statistical tests were conducted with α=0.05.

| **Supplemental Table 2**. Participant characteristics by prior participation in a clinical trial | | | | |  |
| --- | --- | --- | --- | --- | --- |
|  | **Prior participation** | | | |  |
|  | Yes (N=91, 14.2%) | | No (N=552, 85.9%) | |  |
|  | N | % | N | % | p |
| Pregnancy status |  |  |  |  | 0.99 |
| Pregnant | 26 | 28.6 | 104 | 18.8 |  |
| Postpartum | 65 | 71.4 | 448 | 81.2 |  |
| Breastfeeding behavior (among postpartum only) |  |  |  |  | 0.57 |
| Ever breastfed | 62 | 96.9 | 438 | 98.0 |  |
| Never breastfed | 2 | 3.1 | 9 | 2.0 |  |
| Age (years) |  |  |  |  | 0.24 |
| 18-24 | 7 | 7.7 | 61 | 11.1 |  |
| 25-29 | 21 | 23.1 | 131 | 23.7 |  |
| 30-34 | 30 | 33.0 | 214 | 38.8 |  |
| 35-44 | 33 | 36.3 | 146 | 26.5 |  |
| Mean (SD) | 32.1 | 5.1 | 31.4 | 5.1 |  |
| Race |  |  |  |  | <0.01 |
| White | 69 | 76.7 | 468 | 84.9 |  |
| Black | 6 | 6.7 | 18 | 3.3 |  |
| American Indian/Alaska Native | 3 | 3.3 | 3 | 0.5 |  |
| Asian | 6 | 6.7 | 14 | 2.5 |  |
| Pacific Islander | 1 | 1.1 | 1 | 0.2 |  |
| Other | 3 | 3.3 | 20 | 3.6 |  |
| Multiracial | 2 | 2.2 | 27 | 4.9 |  |
| Ethnicity |  |  |  |  | 0.16 |
| Non-Hispanic | 78 | 86.7 | 492 | 91.3 |  |
| Hispanic | 12 | 13.3 | 47 | 8.7 |  |
| Education |  |  |  |  | 0.13 |
| ≤High school | 11 | 12.1 | 77 | 14.0 |  |
| Some college or trade school | 31 | 34.1 | 212 | 38.4 |  |
| Bachelor's degree | 21 | 23.1 | 154 | 27.9 |  |
| Postgraduate | 28 | 30.8 | 109 | 19.8 |  |
| Household income |  |  |  |  | 0.12 |
| Prefer not to answer or don't know | 4 |  | 24 |  |  |
| <$10,000-<$40,000 | 23 | 26.4 | 133 | 25.2 |  |
| ≥$40,000-<$90,000 | 23 | 26.4 | 206 | 39.0 |  |
| ≥$90,000-<$150,000 | 27 | 31.0 | 127 | 24.1 |  |
| ≥$150,000 | 14 | 16.1 | 62 | 11.7 |  |
| Urbanicity |  |  |  |  | 0.04 |
| Rural | 20 | 22.0 | 88 | 15.9 |  |
| Suburban | 48 | 52.8 | 366 | 66.3 |  |
| Urban | 23 | 25.3 | 98 | 17.8 |  |
| Marital status |  |  |  |  | 0.47 |
| Married or cohabitating | 83 | 91.2 | 515 | 93.3 |  |
| Single | 8 | 8.8 | 37 | 6.7 |  |
| Chronic medical condition |  |  |  |  | 0.62 |
| No | 31 | 34.1 | 203 | 36.8 |  |
| Yes | 60 | 65.9 | 349 | 63.2 |  |
| Gravidity |  |  |  |  | 0.41 |
| Primigravida | 24 | 26.7 | 170 | 31.0 |  |
| Multigravida | 66 | 73.3 | 378 | 69.0 |  |
| History of non-live birth |  |  |  |  | 0.29 |
| No | 45 | 50.0 | 306 | 55.9 |  |
| Yes | 45 | 50.0 | 241 | 44.1 |  |
| General health |  |  |  |  | 0.66 |
| Poor or fair | 6 | 6.6 | 30 | 5.4 |  |
| Good | 28 | 30.8 | 180 | 32.7 |  |
| Very Good | 37 | 40.7 | 194 | 35.2 |  |
| Excellent | 20 | 22.0 | 147 | 26.7 |  |
| Worked in healthcare |  |  |  |  | <0.01 |
| No | 45 | 49.5 | 355 | 64.6 |  |
| Yes | 46 | 50.6 | 195 | 35.5 |  |
| Likely to participate in a clinical trial for a medication during pregnancy |  |  |  |  | <0.01 |
| No | 41 | 46.6 | 374 | 68.3 |  |
| Yes | 47 | 53.4 | 174 | 31.8 |  |
| Likely to participate in a clinical trial for a vaccine during pregnancy |  |  |  |  | <0.01 |
| No | 51 | 56.0 | 434 | 78.6 |  |
| Yes | 40 | 44.0 | 118 | 21.4 |  |
| Previous participation in pregnancy or while breastfeeding |  |  |  |  |  |
| While pregnant | 19 | 20.9 | - | - |  |
| While breastfeeding | 9 | 9.9 | - | - |  |
| Pregnant and breastfeeding | 6 | 6.6 | - | - |  |
| Neither pregnant nor breastfeeding | 57 | 62.6 | - | - |  |

All statistical tests were conducted with α=0.05.

| **Supplemental Table 3**. Participant characteristics by familiarity with clinical trials | | | | | | | |
| --- | --- | --- | --- | --- | --- | --- | --- |
|  | Little (n=91, 14%) | | Moderate (n=273, 42%) | | Substantial (n=285, 44%) | |  |
| Characteristic | N | % | N | % | N | % | p |
| Pregnancy status |  |  |  |  |  |  | <0.01 |
| Pregnant | 29 | 31.9 | 47 | 17.2 | 55 | 19.3 |  |
| Postpartum | 62 | 68.1 | 226 | 82.8 | 230 | 80.7 |  |
| Breastfeeding behavior (among postpartum only) |  |  |  |  |  |  | 0.57 |
| Ever breastfed | 61 | 98.4 | 220 | 97.4 | 225 | 98.7 |  |
| Never breastfed | 1 | 1.6 | 6 | 2.7 | 3 | 1.3 |  |
| Age (years) |  |  |  |  |  |  | <0.01 |
| 18-24 | 17 | 18.7 | 33 | 12.1 | 21 | 7.4 |  |
| 25-29 | 31 | 34.1 | 67 | 24.5 | 52 | 18.3 |  |
| 30-34 | 33 | 36.3 | 91 | 33.3 | 121 | 42.5 |  |
| 35-44 | 10 | 11.0 | 82 | 30.0 | 91 | 31.9 |  |
| Mean (SD) | 29.2 | 4.6 | 31.3 | 5.3 | 32.3 | 4.9 | <0.01 |
| Race |  |  |  |  |  |  | 0.15 |
| White | 73 | 80.2 | 235 | 86.4 | 235 | 82.8 |  |
| Black | 2 | 2.2 | 9 | 3.3 | 12 | 4.2 |  |
| American Indian/Alaska Native | 1 | 1.1 | 1 | 0.4 | 4 | 1.4 |  |
| Asian | 1 | 1.1 | 8 | 2.9 | 11 | 3.9 |  |
| Pacific Islander | 0 | 0.0 | 1 | 0.4 | 1 | 0.4 |  |
| Other | 8 | 8.8 | 4 | 1.5 | 11 | 3.9 |  |
| Multiracial | 6 | 6.6 | 14 | 5.2 | 10 | 3.5 |  |
| Ethnicity |  |  |  |  |  |  | 0.19 |
| Non-Hispanic | 77 | 85.6 | 246 | 91.8 | 254 | 91.4 |  |
| Hispanic | 13 | 14.4 | 22 | 8.2 | 24 | 8.6 |  |
| Education |  |  |  |  |  |  | <0.01 |
| ≤High school | 32 | 35.2 | 35 | 12.8 | 21 | 7.4 |  |
| Some college or trade school | 46 | 50.6 | 103 | 37.7 | 97 | 34.0 |  |
| Bachelor's degree | 11 | 12.1 | 87 | 31.9 | 80 | 28.1 |  |
| Postgraduate | 2 | 2.2 | 48 | 17.6 | 87 | 30.5 |  |
| Household income |  |  |  |  |  |  | <0.01 |
| Prefer not to answer or don't know | 5 |  | 18 |  |  |  |  |
| <$10,000-<$40,000 | 40 | 46.5 | 57 | 22.4 | 60 | 21.6 |  |
| ≥$40,000-<$90,000 | 33 | 38.4 | 113 | 44.3 | 85 | 30.6 |  |
| ≥$90,000-<$150,000 | 10 | 11.6 | 64 | 25.1 | 81 | 29.1 |  |
| ≥$150,000 | 3 | 3.5 | 21 | 8.2 | 52 | 18.7 |  |
| Urbanicity |  |  |  |  |  |  | 0.06 |
| Rural | 18 | 19.8 | 47 | 17.2 | 43 | 15.1 |  |
| Suburban | 47 | 51.7 | 181 | 66.3 | 190 | 66.7 |  |
| Urban | 26 | 28.6 | 45 | 16.5 | 52 | 18.3 |  |
| Marital status |  |  |  |  |  |  | 0.10 |
| Married or cohabitating | 80 | 87.9 | 258 | 94.5 | 266 | 93.3 |  |
| Single | 11 | 12.1 | 15 | 5.5 | 19 | 6.7 |  |
| Chronic medical condition |  |  |  |  |  |  | 0.05 |
| No | 28 | 30.8 | 89 | 32.6 | 118 | 41.4 |  |
| Yes | 63 | 69.2 | 184 | 67.4 | 167 | 58.6 |  |
| Gravidity |  |  |  |  |  |  | 0.55 |
| Primigravida | 23 | 25.8 | 87 | 32.0 | 85 | 30.0 |  |
| Multigravida | 66 | 74.2 | 185 | 68.0 | 198 | 70.0 |  |
| History of non-live birth |  |  |  |  |  |  | 0.67 |
| No | 47 | 52.8 | 154 | 56.6 | 150 | 53.2 |  |
| Yes | 42 | 47.2 | 118 | 43.4 | 132 | 46.8 |  |
| Worked in healthcare |  |  |  |  |  |  | <0.01 |
| No | 69 | 75.8 | 195 | 71.7 | 140 | 49.3 |  |
| Yes | 22 | 24.2 | 77 | 28.3 | 144 | 50.7 |  |
| Prior participation in clinical trial |  |  |  |  |  |  | <0.01 |
| No | 83 | 96.5 | 250 | 92.9 | 214 | 75.6 |  |
| Yes | 3 | 3.5 | 19 | 7.1 | 69 | 24.4 |  |
| General health |  |  |  |  |  |  | <0.01 |
| Poor or fair | 3 | 3.3 | 17 | 6.2 | 14 | 4.9 |  |
| Good | 43 | 47.3 | 86 | 31.5 | 82 | 28.9 |  |
| Very Good | 27 | 29.7 | 113 | 41.4 | 96 | 33.8 |  |
| Excellent | 18 | 19.8 | 57 | 20.9 | 92 | 32.4 |  |
| Excludes n=5 individuals who reported they had never heard of clinical trials.  All statistical tests were conducted with α=0.05. | | | | | | | |
|  |  |  |  |  |  |  |  |

| **Supplemental Table 4.** Motivators and deterrents and respective domains for participation in a clinical trial in pregnancy or while breastfeeding | | | | | |
| --- | --- | --- | --- | --- | --- |
|  |  | **Pregnancy** | | **Breastfeeding** | |
| **Motivator** | **Domain** | N | % | N | % |
| Help patients with same medical problem* | Altruism |  |  |  |  |
| Not at all important |  | 16 | 3.8 | 16 | 5.2 |
| Slightly important |  | 40 | 9.6 | 41 | 13.2 |
| Moderately important |  | 112 | 26.9 | 89 | 28.7 |
| Very important |  | 147 | 35.3 | 83 | 26.8 |
| Extremely important |  | 102 | 24.5 | 81 | 26.1 |
| Contribute to research | Altruism |  |  |  |  |
| Not at all important |  | 47 | 7.2 | 54 | 10.7 |
| Slightly important |  | 85 | 13.0 | 96 | 19.1 |
| Moderately important |  | 171 | 26.2 | 144 | 28.6 |
| Very important |  | 184 | 28.1 | 103 | 20.4 |
| Extremely important |  | 167 | 25.5 | 107 | 21.2 |
| Gratitude for medical community | Altruism |  |  |  |  |
| Not at all important |  | 168 | 25.7 | 177 | 35.1 |
| Slightly important |  | 148 | 22.6 | 111 | 22.0 |
| Moderately important |  | 180 | 27.5 | 108 | 21.4 |
| Very important |  | 83 | 12.7 | 49 | 9.7 |
| Extremely important |  | 75 | 11.5 | 59 | 11.7 |
| Help find new treatments | Altruism |  |  |  |  |
| Not at all important |  | 39 | 6.0 | 36 | 7.1 |
| Slightly important |  | 88 | 13.5 | 90 | 17.9 |
| Moderately important |  | 176 | 26.9 | 124 | 24.6 |
| Very important |  | 191 | 29.2 | 138 | 27.4 |
| Extremely important |  | 160 | 24.5 | 116 | 23.0 |
| Severity of my medical condition* | Personal medical benefit |  |  |  |  |
| Not at all important |  | 7 | 1.7 | 6 | 1.9 |
| Slightly important |  | 20 | 4.8 | 22 | 7.1 |
| Moderately important |  | 102 | 24.5 | 84 | 27.1 |
| Very important |  | 144 | 34.5 | 96 | 31.0 |
| Extremely important |  | 144 | 34.5 | 102 | 32.9 |
| Obtain information about my disease* | Personal medical benefit |  |  |  |  |
| Not at all important |  | 30 | 7.2 | 31 | 10.0 |
| Slightly important |  | 52 | 12.5 | 50 | 16.1 |
| Moderately important |  | 126 | 30.2 | 86 | 27.7 |
| Very important |  | 135 | 32.4 | 79 | 25.5 |
| Extremely important |  | 74 | 17.8 | 64 | 20.7 |
| Improve own treatment* | Personal medical benefit |  |  |  |  |
| Not at all important |  | 13 | 3.1 | 11 | 3.6 |
| Slightly important |  | 30 | 7.2 | 31 | 10.0 |
| Moderately important |  | 79 | 18.9 | 69 | 22.3 |
| Very important |  | 167 | 40.1 | 108 | 34.8 |
| Extremely important |  | 128 | 30.7 | 91 | 29.4 |
| Receive different treatments* | Personal medical benefit |  |  |  |  |
| Not at all important |  | 21 | 5.0 | 19 | 6.1 |
| Slightly important |  | 47 | 11.3 | 28 | 9.0 |
| Moderately important |  | 114 | 27.3 | 94 | 30.3 |
| Very important |  | 127 | 30.5 | 96 | 31.0 |
| Extremely important |  | 108 | 25.9 | 73 | 23.6 |
| Active role in my healthcare | Personal medical benefit |  |  |  |  |
| Not at all important |  | 86 | 13.2 | 90 | 17.9 |
| Slightly important |  | 121 | 18.5 | 111 | 22.0 |
| Moderately important |  | 211 | 32.3 | 146 | 29.0 |
| Very important |  | 136 | 20.8 | 89 | 17.7 |
| Extremely important |  | 100 | 15.3 | 68 | 13.5 |
| Potential health benefit | Personal medical benefit |  |  |  |  |
| Not at all important |  | 23 | 3.5 | 23 | 4.6 |
| Slightly important |  | 49 | 7.5 | 47 | 9.3 |
| Moderately important |  | 159 | 24.3 | 140 | 27.8 |
| Very important |  | 236 | 36.1 | 158 | 31.4 |
| Extremely important |  | 187 | 28.6 | 136 | 27.0 |
| Extra medical attention | Personal medical benefit |  |  |  |  |
| Not at all important |  | 122 | 18.7 | 120 | 23.8 |
| Slightly important |  | 106 | 16.2 | 95 | 18.9 |
| Moderately important |  | 148 | 22.6 | 114 | 22.6 |
| Very important |  | 154 | 23.6 | 93 | 18.5 |
| Extremely important |  | 124 | 19.0 | 82 | 16.3 |
| Receive findings | Personal medical benefit |  |  |  |  |
| Not at all important |  | 100 | 15.3 | 107 | 21.2 |
| Slightly important |  | 140 | 21.4 | 100 | 19.8 |
| Moderately important |  | 167 | 25.5 | 123 | 24.4 |
| Very important |  | 142 | 21.7 | 96 | 19.1 |
| Extremely important |  | 105 | 16.1 | 78 | 15.5 |
| Financial compensation | Financial |  |  |  |  |
| Not at all important |  | 126 | 19.3 | 106 | 21.0 |
| Slightly important |  | 131 | 20.0 | 121 | 24.0 |
| Moderately important |  | 172 | 26.3 | 113 | 22.4 |
| Very important |  | 100 | 15.3 | 70 | 13.9 |
| Extremely important |  | 125 | 19.1 | 94 | 18.7 |
| Doctor endorsement | Support network approval |  |  |  |  |
| Not at all important |  | 85 | 13.0 | 84 | 16.7 |
| Slightly important |  | 123 | 18.8 | 101 | 20.0 |
| Moderately important |  | 214 | 32.7 | 162 | 32.1 |
| Very important |  | 146 | 22.3 | 93 | 18.5 |
| Extremely important |  | 86 | 13.2 | 64 | 12.7 |
| Family/friend endorsement | Support network approval |  |  |  |  |
| Not at all important |  | 213 | 32.6 | 184 | 36.5 |
| Slightly important |  | 187 | 28.6 | 144 | 28.6 |
| Moderately important |  | 168 | 25.7 | 109 | 21.6 |
| Very important |  | 48 | 7.3 | 37 | 7.3 |
| Extremely important |  | 38 | 5.8 | 30 | 6.0 |
| Partner endorsement | Support network approval |  |  |  |  |
| Not at all important |  | 155 | 23.7 | 131 | 26.0 |
| Slightly important |  | 168 | 25.7 | 119 | 23.6 |
| Moderately important |  | 188 | 28.8 | 145 | 28.8 |
| Very important |  | 91 | 13.9 | 62 | 12.3 |
| Extremely important |  | 52 | 8.0 | 47 | 9.3 |
| **Deterrent** |  |  |  |  |  |
| Possible side effects on me | Safety concerns for oneself |  |  |  |  |
| Not at all important |  | 13 | 2.0 | 18 | 3.6 |
| Slightly important |  | 54 | 8.3 | 74 | 14.7 |
| Moderately important |  | 122 | 18.7 | 112 | 22.2 |
| Very important |  | 208 | 31.8 | 118 | 23.4 |
| Extremely important |  | 257 | 39.3 | 182 | 36.1 |
| Possible risk to future fertility | Safety concerns for oneself |  |  |  |  |
| Not at all important |  | 49 | 7.5 | 57 | 11.3 |
| Slightly important |  | 31 | 4.7 | 28 | 5.6 |
| Moderately important |  | 63 | 9.6 | 57 | 11.3 |
| Very important |  | 99 | 15.1 | 75 | 14.9 |
| Extremely important |  | 412 | 63.0 | 287 | 56.9 |
| Possible side effects on baby | Safety concerns for fetus/baby |  |  |  |  |
| Not at all important |  | 4 | 0.6 | 3 | 0.6 |
| Slightly important |  | 7 | 1.1 | 4 | 0.8 |
| Moderately important |  | 15 | 2.3 | 13 | 2.6 |
| Very important |  | 47 | 7.2 | 43 | 8.5 |
| Extremely important |  | 581 | 88.8 | 441 | 87.5 |
| Possible long-term effects on child | Safety concerns for fetus/baby |  |  |  |  |
| Not at all important |  | 6 | 0.9 | 3 | 0.6 |
| Slightly important |  | 32 | 4.9 | 11 | 2.2 |
| Moderately important |  | 71 | 10.9 | 49 | 9.7 |
| Very important |  | 118 | 18.0 | 80 | 15.9 |
| Extremely important |  | 427 | 65.3 | 361 | 71.6 |
| Possible risk to pregnancy | Safety concerns for fetus/baby |  |  |  |  |
| Not at all important |  | 8 | 1.2 | - | - |
| Slightly important |  | 6 | 0.9 | - | - |
| Moderately important |  | 18 | 2.8 | - | - |
| Very important |  | 50 | 7.7 | - | - |
| Extremely important |  | 572 | 87.5 | - | - |
| May cause distress/pain to fetus | Safety concerns for fetus/baby |  |  |  |  |
| Not at all important |  | 49 | 7.5 | - | - |
| Slightly important |  | 34 | 5.2 | - | - |
| Moderately important |  | 97 | 14.8 | - | - |
| Very important |  | 132 | 20.2 | - | - |
| Extremely important |  | 342 | 52.3 | - | - |
| Effects on milk production | Safety concerns for fetus/baby |  |  |  |  |
| Not at all important |  | - | - | 2 | 0.4 |
| Slightly important |  | - | - | 13 | 2.6 |
| Moderately important |  | - | - | 49 | 9.7 |
| Very important |  | - | - | 95 | 18.9 |
| Extremely important |  | - | - | 345 | 68.5 |
| Interrupt breastfeeding | Safety concerns for fetus/baby |  |  |  |  |
| Not at all important |  | - | - | 4 | 0.8 |
| Slightly important |  | - | - | 21 | 4.2 |
| Moderately important |  | - | - | 46 | 9.1 |
| Very important |  | - | - | 114 | 22.6 |
| Extremely important |  | - | - | 319 | 63.3 |
| Time commitment | Logistics challenges |  |  |  |  |
| Not at all important |  | 164 | 25.1 | 154 | 30.6 |
| Slightly important |  | 191 | 29.2 | 138 | 27.4 |
| Moderately important |  | 161 | 24.6 | 120 | 23.8 |
| Very important |  | 85 | 13.0 | 51 | 10.1 |
| Extremely important |  | 53 | 8.1 | 41 | 8.1 |
| Conflict with work | Logistics challenges |  |  |  |  |
| Not at all important |  | 239 | 36.5 | 212 | 42.1 |
| Slightly important |  | 152 | 23.2 | 111 | 22.0 |
| Moderately important |  | 124 | 19.0 | 82 | 16.3 |
| Very important |  | 85 | 13.0 | 56 | 11.1 |
| Extremely important |  | 54 | 8.3 | 43 | 8.5 |
| Lack of transportation | Logistics challenges |  |  |  |  |
| Not at all important |  | 459 | 70.2 | 371 | 73.6 |
| Slightly important |  | 73 | 11.2 | 55 | 10.9 |
| Moderately important |  | 50 | 7.7 | 32 | 6.4 |
| Very important |  | 35 | 5.4 | 24 | 4.8 |
| Extremely important |  | 37 | 5.7 | 22 | 4.4 |
| Lack of childcare | Logistics challenges |  |  |  |  |
| Not at all important |  | 203 | 31.0 | 155 | 30.8 |
| Slightly important |  | 131 | 20.0 | 111 | 22.0 |
| Moderately important |  | 106 | 16.2 | 90 | 17.9 |
| Very important |  | 108 | 16.5 | 70 | 13.9 |
| Extremely important |  | 106 | 16.2 | 78 | 15.5 |
| Friend/family disapproval | Support network disapproval |  |  |  |  |
| Not at all important |  | 357 | 54.6 | 291 | 57.7 |
| Slightly important |  | 144 | 22.0 | 110 | 21.8 |
| Moderately important |  | 92 | 14.1 | 66 | 13.1 |
| Very important |  | 31 | 4.7 | 18 | 3.6 |
| Extremely important |  | 30 | 4.6 | 19 | 3.8 |
| Partner disapproval | Support network disapproval |  |  |  |  |
| Not at all important |  | 197 | 30.1 | 158 | 31.4 |
| Slightly important |  | 105 | 16.1 | 86 | 17.1 |
| Moderately important |  | 125 | 19.1 | 103 | 20.4 |
| Very important |  | 122 | 18.7 | 73 | 14.5 |
| Extremely important |  | 105 | 16.1 | 84 | 16.7 |
| Don't want to be experimented upon | Uncomfortable with experimentation |  |  |  |  |
| Not at all important |  | 202 | 30.9 | 181 | 35.9 |
| Slightly important |  | 145 | 22.2 | 125 | 24.8 |
| Moderately important |  | 146 | 22.3 | 101 | 20.0 |
| Very important |  | 79 | 12.1 | 44 | 8.7 |
| Extremely important |  | 82 | 12.5 | 53 | 10.5 |
| Don't like hospitals/doctor's offices | Dislike medical interventions |  |  |  |  |
| Not at all important |  | 348 | 53.2 | 309 | 61.3 |
| Slightly important |  | 121 | 18.5 | 80 | 15.9 |
| Moderately important |  | 107 | 16.4 | 70 | 13.9 |
| Very important |  | 33 | 5.1 | 13 | 2.6 |
| Extremely important |  | 45 | 6.9 | 32 | 6.4 |
| Concern about having blood drawn | Dislike medical interventions |  |  |  |  |
| Not at all important |  | 476 | 72.8 | 366 | 72.6 |
| Slightly important |  | 79 | 12.1 | 66 | 13.1 |
| Moderately important |  | 53 | 8.1 | 38 | 7.5 |
| Very important |  | 22 | 3.4 | 15 | 3.0 |
| Extremely important |  | 24 | 3.7 | 19 | 3.8 |
| Concern about undergoing medical tests | Dislike medical interventions |  |  |  |  |
| Not at all important |  | 368 | 56.3 | 310 | 61.5 |
| Slightly important |  | 125 | 19.1 | 88 | 17.5 |
| Moderately important |  | 87 | 13.3 | 63 | 12.5 |
| Very important |  | 40 | 6.1 | 21 | 4.2 |
| Extremely important |  | 34 | 5.2 | 22 | 4.4 |
| Lack of trust in pharmaceutical companies | Lack of trust in pharmaceutical companies |  |  |  |  |
| Not at all important |  | 77 | 11.8 | 92 | 18.3 |
| Slightly important |  | 115 | 17.6 | 112 | 22.2 |
| Moderately important |  | 165 | 25.2 | 129 | 25.6 |
| Very important |  | 119 | 18.2 | 63 | 12.5 |
| Extremely important |  | 178 | 27.2 | 108 | 21.4 |
| Asterisks denote items that were only queried among those who specified they had a chronic medical condition. | | | | | |

| **Supplemental Table 5**. Measures of internal consistency for motivator and deterrent domains for participation in clinical trials in pregnancy or while breastfeeding | | |
| --- | --- | --- |
|  | Chronbach's alpha | |
| **Motivators** | Pregnancy | Breastfeeding |
| Altruism | 0.88 | 0.90 |
| Personal medical benefit | 0.85 | 0.89 |
| Financial | NA | NA |
| Support network approval | 0.78 | 0.80 |
| **Deterrents** |  |  |
| Safety concerns for oneself | 0.56 | 0.51 |
| Safety concerns for fetus or baby | 0.68 | 0.79 |
| Logistics challenges | 0.73 | 0.77 |
| Support network disapproval | 0.69 | 0.68 |
| Uncomfortable with experimentation | NA | NA |
| Dislike of medical interventions | 0.77 | 0.80 |
| Distrust in pharmaceutical companies | NA | NA |
| NA is denoted when domains were comprised of a single item | | |

| **Supplemental Table 6**. Proportions of individuals that endorsed motivator and deterrent domain(s) of clinical trial participation by likelihood of participation in a clinical trial for a medication or vaccine in pregnancy (n=654) or while breastfeeding (n=504) | | | | | | | | | | |
| --- | --- | --- | --- | --- | --- | --- | --- | --- | --- | --- |
|  | *In pregnancy* | | | | | | | | | |
|  | Overall (n=654) | | Participation in a clinical trial for a medication^a^ | | | | Participation in a clinical trial for a vaccine | | | |
|  |  |  | Likely  (n=225, 35%) | | Not likely (422, 65%) | | Likely  (n=160, 24%) | | Not likely  (n=494, 76%) | |
|  | N | % | N | % | N | % | N | % | N | % |
| *Motivators* |  |  |  |  |  |  |  |  |  |  |
| Personal medical benefit | 561 | 85.8 | 206 | 91.6 | 349 | 82.7 | 149 | 93.1 | 412 | 83.4 |
| Altruism | 457 | 69.9 | 198 | 88.0 | 253 | 60.0 | 145 | 90.6 | 312 | 63.2 |
| Financial compensation | 225 | 34.4 | 85 | 37.8 | 137 | 32.5 | 53 | 33.1 | 172 | 34.8 |
| Support network endorsement | 287 | 43.9 | 114 | 50.7 | 170 | 40.3 | 84 | 52.5 | 203 | 41.1 |
| *Deterrents* |  |  |  |  |  |  |  |  |  |  |
| Safety concerns for oneself | 573 | 87.6 | 183 | 81.3 | 385 | 91.2 | 125 | 78.1 | 448 | 90.7 |
| Safety concerns for fetus/baby | 640 | 97.9 | 216 | 96.0 | 417 | 98.8 | 152 | 95.0 | 488 | 98.8 |
| Logistics challenges | 308 | 47.1 | 98 | 43.6 | 208 | 49.3 | 70 | 43.8 | 238 | 48.2 |
| Support network disapproval | 234 | 35.8 | 64 | 28.4 | 167 | 39.6 | 45 | 28.1 | 189 | 38.3 |
| Uncomfortable with experimentation | 161 | 24.6 | 32 | 14.2 | 127 | 30.1 | 11 | 6.9 | 150 | 30.4 |
| Dislike of medical interventions | 130 | 19.9 | 32 | 14.2 | 97 | 23.0 | 17 | 10.6 | 113 | 22.9 |
| Distrust in pharma | 297 | 45.4 | 75 | 33.3 | 219 | 51.9 | 39 | 24.4 | 258 | 52.2 |
|  | *While breastfeeding* | | | | | | | | | |
|  | Overall (n=504) | | Participation in a clinical trial for a medication | | | | Participation in a clinical trial for a vaccine | | | |
|  |  |  | Likely (206, 41%) | | Not likely  (n=298, 59%) | | Likely  (n=210, 42%) | | Not likely  (n=294, 58%) | |
|  | N | % | N | % | N | % | N | % | N | % |
| *Motivators* |  |  |  |  |  |  |  |  |  |  |
| Personal medical benefit | 396 | 78.6 | 178 | 86.4 | 218 | 73.2 | 183 | 87.1 | 213 | 72.5 |
| Altruism | 313 | 62.1 | 171 | 83.0 | 142 | 47.7 | 171 | 81.4 | 142 | 48.3 |
| Financial compensation | 164 | 32.5 | 76 | 36.9 | 88 | 29.5 | 67 | 31.9 | 97 | 33.0 |
| Support network endorsement | 206 | 40.9 | 94 | 45.6 | 112 | 37.6 | 101 | 48.1 | 105 | 35.7 |
| *Deterrents* |  |  |  |  |  |  |  |  |  |  |
| Safety concerns for oneself | 418 | 82.9 | 146 | 70.9 | 272 | 91.3 | 154 | 73.3 | 264 | 89.8 |
| Safety concerns for fetus/baby | 490 | 97.2 | 196 | 95.2 | 294 | 98.7 | 201 | 95.7 | 289 | 98.3 |
| Logistics challenges | 209 | 41.5 | 81 | 39.3 | 128 | 43.0 | 78 | 37.1 | 131 | 44.6 |
| Support network disapproval | 161 | 31.9 | 51 | 24.8 | 110 | 36.9 | 54 | 25.7 | 107 | 36.4 |
| Uncomfortable with experimentation | 97 | 19.3 | 14 | 6.8 | 83 | 27.9 | 10 | 4.8 | 87 | 29.6 |
| Dislike of medical interventions | 77 | 15.3 | 15 | 7.3 | 62 | 20.8 | 19 | 9.1 | 58 | 19.7 |
| Distrust in pharma | 171 | 33.9 | 35 | 17.0 | 136 | 45.6 | 30 | 14.3 | 141 | 48.0 |
| ^a^Excludes n=7 individuals with missing responses | | |  |  |  |  |  |  |  |  |
